# Fungal β-glucan instructed miR-32-5p modulates Dectin-1 signaling mediated inflammation, reactive oxygen species and apoptosis through polarization of “M2a-like” macrophage in *Candida* colitis

**DOI:** 10.1080/21505594.2025.2514789

**Published:** 2025-06-06

**Authors:** Liu Yang, Chengcheng Liu, Hanyu Zhu, Zixu Wang, Qinai Luo, Yuzhe Huang, Hao Wang, Min Hu, Jing Shao

**Affiliations:** a Laboratory of Infection and Immunity, College of Integrated Chinese and Western Medicine (College of Life Science), Anhui University of Chinese Medicine, Hefei, Anhui, P. R. China; b Department of pathology, College of Integrated Chinese and Western Medicine (College of Life Science), Anhui University of Chinese Medicine, Hefei, Anhui, P. R. China; c Department of Pharmacy, Anhui University of Chinese Medicine, Hefei, Anhui, P. R. China; d Anhui Province Key Laboratory of Meridian Viscera Correlationship, Anhui University of Chinese Medicine, Hefei, Anhui, P. R. China; e Institute of Integrated Traditional Chinese and Western Medicine, Anhui Academy of Chinese Medicine, Hefei, Anhui, P. R. China

**Keywords:** Ulcerative colitis, *Candida albicans*, β-glucan, Dectin-1, miR-32-5p, oxidative stress

## Abstract

Ulcerative colitis (UC) is a chronic and easy-to-relapse intestinal disease characterized by colon inflammation and microbial dysbiosis. *Candida albicans* is the most common fungal resident in the human gut. Overgrowth of *C. albicans* has been linked to the aggravation of UC. Previously, we demonstrated that miR-32-5p was the most differentially expressed microRNA in DSS-induced colitis model supplemented with *C. albicans* and might be a potential target in the treatment of *Candida* colitis. However, the underlying pathogenic and therapeutic mechanisms remain unclear. Here, we firstly used the ITS technique to analyse intestinal mycobiota. The miR-32-5p adenovirus in company with extracted *Candida* cell wall β-glucan were then employed to monitor the impacts of miR-32-5p and fungal β-glucan on the severity of *Candida* colitis. Subsequently, gene silencing together with inhibitors of reactive oxygen species (ROS) and apoptosis was used to survey the modulation of miR-32-5p on macrophage polarization. According to these results, *C. albicans* became the dominant intestinal fungal species in *Candida* colitis model. *Candida* β-glucan could instruct miR-32-5p expression to affect the severity of *Candida* colitis in a concentration-dependent manner. Interestingly, high fungal β-glucan with pro-inflammatory effects hindered further increases in gut inflammation. Further analysis showed that overexpression of miR-32-5p could effectively inhibit inflammation and apoptosis and enhance phagocytosis and ROS production through Dectin-1 signalling in macrophages. A panel of representative gene expressions verified the polarization of the M2-like phenotype induced by miR-32-5p. Mechanistically, our results reveal the therapeutic potential of miR-32-5p in the amelioration of *Candida* colitis.

## Introduction

Ulcerative colitis (UC) is an idiopathic form of chronic inflammatory bowel disease (IBD) and characterized by persistent inflammation of the rectum and colon to a variable extent. By 2023, the morbidity of UC was estimated to be 5 million worldwide, and its incidence is still increasing. Although the pathogenesis of UC is not fully understood, individuals with inappropriate immune responses and intestinal dysbiosis are predisposed to this disease. Although the role of bacterial flora (microbiota) in UC has been extensively studied [1], the role of fungal flora (mycobiota) in the occurrence and development of UC remains largely unexplored.

*Candida albicans* is the most common dimorphic fungal pathogen that resides in the human gut and has been listed as a “critical fungal priority pathogen” by the World Health Organization (https://www.who.int/publications/i/item/9789240060241). When the host immune system is compromised, *C. albicans* tends to cause mucosal or deep-seated infections (i.e. oropharyngeal candidiasis and candidemia). A previous report showed that *C. albicans* (91%) was the most prevalent *Candida* spp. followed by *C. glabrata* (6.7%) and *C. incospicua* (1.6%) among the fungal strains isolated from the colon of UC patients [2]. Several clinical reports have demonstrated that the genus *Candida* was found in 28–32% of UC patients [3,4], and a very marked increase in *C. albicans* was observed in patients with stenosing behaviour compared to those with the inflammatory phenotype [5]. A recent report showed that the relative abundance of the genus *Candida* in patients with clinical activity increased 3.5 fold compared with that during remission [6]. Furthermore, Yan et al. constructed a cultivated gut fungi catalogue (760 genomes, 206 species) from the faeces of healthy individuals and found that *C. albicans* overabundance was prevalent in the stools of unhealthy individuals, especially IBD patients, in Chinese and non-Chinese populations [7]. Fungal profiles have been regarded as markers after treatment with anti-TNF-α in IBD patients [8], and antifungal therapy, such as fluconazole, can be beneficial in the restoration and healing of colonic damages in UC patients [2,4]. Although this fungus does not naturally colonize the mouse gut, our sequencing results demonstrated that exogenous *C. albicans* could cause significant intestinal dysbiosis of microbiota and mycobiota [9]. Therefore, *C. albicans* may be a potential pathogenic target in UC therapy.

Antifungal immune mechanisms against *C. albicans* have been widely explored in multiple pattern recognition receptor (PRR) associated pathways [10–12]. The C-type lectin receptor Dectin-1 which is broadly distributed in myeloid cells can recognize the cell wall β-glucan of *C. albcians* [13], and specifically stimulate downstream IgA- and Th17-mediated mucosal immune responses [14,15], which are verified to be critical antifungal responses to excessive *C. albicans* in UC colons [16,17]. Iliev et al. reported that mice lacking Dectin-1 became susceptible to DSS-induced colitis due to an altered response to indigenous fungi and identified a potential correlation of CLEC7A (encoding Dectin-1) single-nucleotide polymorphism rs2078178 in patients with medially refractory UC [18]. After engagement with *C. albcians*, Dectin-1 receptor forms a “phagocytic synapse” [19], then recruits and phosphorylates the adaptor Syk to initiate the downstream signalling cascades, eventually triggering a set of pro-inflammatory cytokines including TNF-α, IL-1β, IL-6, and IL-17 and the generation of reactive oxygen species (ROS) [20]. Currently, the management of UC has shifted from symptom-free daily living to mucosal healing. Macrophages are a pivotal member of the immune system and can make a function on the regeneration and repair of colonic ulcers [21]. M2 macrophages are commonly divided into four categories: M2a (alternatively activated macrophages), M2b (type 2 macrophages), M2c (deactivated macrophages) and M2d [22]. Since M2a macrophages are usually involved in tissue repair, angiogenesis, and fibrosis, they have been referred to as “wound-healing macrophages” [23]. In terms of high inflammatory levels in UC colon; however, the proper restriction or reversion of intestinal inflamed environment via M2 phenotypes is still unexplored during Dectin-1 mediated antifungal reactions.

MicroRNAs (miRNAs) are a bunch of 18–23 nucleotide long non-coding RNAs that are involved in the regulation of post-transcriptional gene expression. Liu et al. discovered that the host could shape the gut microbiota via faecal miRNA, which is essential for homoeostasis of the intestinal microbiota [24]. In a previous study, we sequenced and profiled intestinal miRNAs and identified miR-32-5p as a potential target that was significantly downregulated in a DSS-induced colitis mouse model in the presence of *C. albicans* [25]. After screening and comparison, we noticed that miR-32-5p might be a characteristic miRNA in this model, as we did not find clues on the identification of miR-32-5p in UC or fungal infections [26–28]. Interestingly, our results showed that increased miR-32-5p markedly inhibited the fungal capacity of *C. albicans* with enhanced expression of Dectin-1 and downregulation of pro-inflammatory cytokines (i.e. TNF-α, IL-1β and IL-17) in Caco2 [25]. These experimental signs indicate a correlation between miR-32-5p with Dectin-1 mediated M2 macrophages.

In this study, we aim to explore the amelioration mechanism of fungal cell wall β-glucan instructed miR-32-5p in the treatment of DSS-induced colitis with *C. albicans* supplementation, by which activated Decint-1 associated signalling modulates intestinal ROS and inflammation through polarization of “M2a-like” macrophages.

## Materials and methods

### Strain propagation

*C. albicans* SC5314 was kindly donated by Prof. Yuanying Jiang of the Second Military Medical University (Shanghai, China). The clinical strains *C. albicans* Z5214, Z4935 and Z5172 were provided by the Dermatology Department of the First Affiliated Hospital of Anhui University of Chinese Medicine (Hefei, China), and were used in our previous reports [29,30]. The strains were seeded in liquid Sabouraud medium (HB0379, Hopebio, Qingdao, China) at 37°C overnight and collected at 3,000 g (Leiboer Medical Devices, Beijing, China). The fungal cells were then propagated in yeast peptone dextrose (YPD) broth (HB5193–1, Hopebio, Qingdao, China) at 37°C for 12–16 h to reach exponential growth stage.

### Cell cultivation

LS174T cells were purchased from iCell Bioscience Inc (Shanghai, China). Caco2 cells were acquire from MeilunBio (Dalian, China). HT29 cells were bought from Pricella Biotechnology (Wuhan, China). NCM460 cells were got from Fenghui Biotechnology (Hunan, China). RAW264.7, THP-1 and HCT116 cells were obtained from the Cell Bank of the Chinese Academy of Sciences (Shanghai, China). All cells were cultured in Dulbecco’s modified Eagle’s medium (DMEM; Gibco, USA) or Roswell Park Memorial Institute (RPMI 1640; Gibco, USA) augmented with 10% foetal bovine serum (FBS; Gibco, USA) and 100 U/mL penicillin/streptomycin (Beyotime, China) at 37°C in 5% CO_2_. NCM460 cells were treated with DSS (20 mg/ml) for 12 h to create an in vitro colitis model [31].

### Internal transcribed spacer (ITS) sequencing

After sacrifice of mice with *Candida* colitis, the faecal samples were collected into sterile EP tubes, placed in dry ice, and immediately sent to Majorbio Bio-Pharm Technology (Shanghai, China) for analysis. The total DNA of the microbial community was extracted according to the instructions of E.Z.N.A.® soil DNA kit (Omega Bio-tek, Norcross, GA, USA). The quality of extracted DNA was monitored using 1% agarose gel electrophoresis. The DNA concentration and purity were determined using NanoDrop2000 (Thermo Scientific, USA). Using DNA samples as templates, the 26S rRNA ITS regions were amplified using the ABI GeneAmp.9700 PCR instrument. The forward and reverse primers (5”−3”) are listed as follows: ITS1F-CTTGGTCATTTAGAGGAAGTAA and ITS4R-TCCTCCGCTTATTGATATGC. The PCR reaction was performed as pre-denaturation at 95°C for 3 mins, followed by 27 cycles of denaturation at 95°C for 30s, annealing at 60°C for 30s, and extension at 72°C for 30s with a stable extension at 72°C for 10 mins as the ending. Sequencing was carried out using the Miseq PE300 platform of Illumina. Similar sequences were clustered into the same operational classification unit (OTU) with a homology up to 97%. The Alpha diversity indices including Abundance-based Coverage Estimator (ACE), Simpsoneven and Shannon indices were assessed. The most commonly used method in principal coordinate analysis (PCoA) β-diversity index was employed to evaluate the differences among fungal communities. The composition and position of the fungal community were evaluated by addition, Venn diagram, community, composition.

### Fungal component preparations

The fungal components of *C. albicans* cells were processed as follows: (1) Heat-killed *C. albicans* (HK-CA): According to previously procedures described [32], *C. albicans* cells were re-suspended in PBS and heated at 100°C for 5 min. The fungal cells were counted to the desired cell density prior to the following experiments. (2) Cell wall of *C. albicans* (CW-CA): The extraction procedures were reported in our previous study [33]. (3) Cell wall β-glucan of *C. albicans* (CWGlu-CA): The extraction procedures were reported in our previous study [33]. (4) Cellular inclusion of *C. albicans* (CI-CA): After the collection of broken CW-CA by centrifugation as described in [33], the remnant supernatants were CI-CA. (5) Glucanase digestion: According to the procedures described [34], a quantity of 1 × 10^8^ cells of *C. albicans* yeasts, 1 mg of CW-CA and 0.5 mg of CWGlu-CA were respectively treated with 1 mg β-1,3-D-glucanase (Yuanye Bio-Tech, Shanghai, China) dissolved in 1 mL sodium acetate buffer (150 mm, pH 5.0; Biosharp, Beijing) at 37°C overnight. The unhydrolyzed particles were preserved by proper centrifugation at 3000 g for 5 min. (6) Supernatant of *C. albicans* (SN-CA): According to the procedures described [35], an inoculum of 1 × 10^8^CFU/mL of *C. albicans* was washed and re-suspended in RPMI1640 medium, cultured overnight, and then centrifuge at 3000 g for 5 min to collect the supernatant.

### Lactate dehydrogenase (LDH) detection

The concentration of LDH in NCM460 cells was measured using an LDH assay kit (Biosharp, Beijing, China), following the manufacturer’s instructions.

### Ethical statement

Animal housing and all experimental procedures were approved by the Animal Ethics Committee of the Anhui University of Chinese Medicine (Animal Ethics Number: AHUM-mouse -2,024,182). The maintenance and treatment of all animals complied with the principles of the Institutional Animal Ethics Committee of the Chinese Center for Disease Control and Prevention and conformed to the Chinese National Guidelines on the Care and Use of Laboratory Animals. The study adhered to the ARRIVE guidelines.

### Colitis establishment

Female C57BL6/J mice (6–8 weeks, 18–22 g) were purchased from Changsheng (License: AHUCM-mouse -2,024,182, Liaoning, China) and housed under a 12/12 h light-dark cycle with ad libitum access to tap water and food. The mice were acclimated for 7–10 days before the experiment. The colitis model was established according to previously published methods [25]. Briefly, a quantity of 2.5% DSS (Zeping, Beijing, China) dissolved in distilled water was drunk daily for free. *C. albicans* SC5314 (1 × 10^8^CUF per mouse), SN-CA (200 μL per mouse), CW-CA (50 mg/kg), CWGlu-CA (20 and 80 mg/kg, CWGlu-CA-20/80), and CWGlu-CA-20 hydrolysed by β-glucanase were intragastrically administrated on day 1, 3, 5, and 7. Curdlan (25 mg/kg) was injected intraperitoneally for two consecutive days from day 1. The sham mice were provided with saline only. The established colitis model was evaluated using the disease activity index (DAI), body weight, and colon length as described previously [25].

### Mir-32-5p adenovirus

Adeno-associated virus 9 was constructed to overexpress mmu-miR-32-5p (AAV9-mmu-miR-32-5p, primer sequence, 5 “-TATTGCACATTACTAAGTTGCA-3”) by GenePharma (Shanghai, China). A volume of 100 μL of AAV9-mmu-miR-32-5p or AAV9-NC negative control was intraperitoneally injected into the mice to a final concentration of 2.5 × 10^12^ V.G/mL two weeks before the induction of colitis model according to the manufacturer’s instruction. The relative expression of mmu-miR-32-5p was analysed using reverse transcription quantitative real-time PCR (RT-qPCR).

### Hematein-Eosin (HE) staining

HE staining was performed using a previously described method with minor adjustments [9]. Briefly, colon tissues were fixed in 10% neutral formalin (Zhenwo Biomedical, Guangzhou, China), embedded in paraffin, sliced into 4 μm-thick section, and stained with HE (Leagene, Beijing, China). Pathological changes were observed using an optical microscope (Olympus BX51, Japan).

### Transfections

As for transfection of miR-32-5p mimics and inhibitors, double-stranded RNA oligonucleotides of mmu-miR-32-5p mimics and inhibitors were purchased from Hanbio (Shanghai, China) and transfected using RNATransMate (E607402–0500, Sangon, Shanghai, China). As per the manufacturer’s instructions, mixture A comprising 20 nmol miRNA mimics or inhibitors, 695 μL of Opti-MEM, and mixture B comprising 42 μL RNATransMate and 660 μL of Opti-MEM were blended for 20 min and then incubated with the cell culture for 24 h. As for transfection of siDectin-1, an inoculum of 3.0 × 10^5^ cells per well was seeded in 6-well plates and cultured in DMEM supplemented with 10% FBS. Three siRNA sequences of the Dectin-1 gene were synthesized by Hanbio and evaluated using PCR before the experiments. As per the manufacturer’s instructions, mixture A comprising 50 nmol siRNA and 250 μL of Opti-MEM and mixture B comprising 5 μL RNATransMate and 250 μL of Opti-MEM were blended for 20 min and then incubated with the cell culture for 24 h.

### FITC-dextran assay

After modelling, the mice were fasted for 4 h. FITC-dextran (0.6 mg/g) was administered orally. After 4 h, 50 μL blood was collected from the eyeball. After standing for 3 h, serum was centrifuged and separated. The serum concentration of FITC-dextran was measured using a multi-functional enzyme labelling instrument (SpectraMaxiD3, MolecularDevices, Shanghai, China) at the excitation and emission wavelengths of 485 nm and 528 nm, respectively.

### Fungal β-glucan exposure

The experimental procedures have been described in our previous study with fewer modifications [33]. Briefly, an inoculum of 1 × 10^6^ cells/mL of *C. albicans* was incubated with caspofungin at 0.005, 0.01, 0.02 μg/mL at 37°C for 24 h. The fungal cells were pooled by centrifugation at 3000× g for 5 min and washed three times with sterile PBS. Subsequently, the fungal pellets were sealed with 2% BSA (9048–46–8, BioFRoxx, Shanghai, China) for 1 h, co-incubated with anti-β-glucan monoclonal antibody (1:300, 400–2, Bioscience Supplies, Australia) at 4°C for 4 h and Cy3 labelled goat anti-mouse IgG (1:200, AB0134, Abways, Shanghai, China) at 4°C for 1 h respectively. After washing with PBS, the collected fungal cells were analysed using flow cytometry (BD FACS Celesta, USA). Fungal cells in isometric RPMI1640 medium were used as controls. A sum of 10 000 fungal cells were screened, and the mean fluorescence intensity (MFI) was monitored using FlowJo v.10 software.

### Phagocytosis

A sum of 9 × 10^5^ CFU/mL of *C. albicans* was co-cultured with caspofungin at the final concentrations of 0.005, 0.01, and 0.02 μg/mL to induce β-glucan exposure for 24 h. After centrifugation to discard the drug-containing medium, an inoculum of 9 × 10^5^ CFU/mL of *C. albicans* was incubated with RAW264.7 cells at 3.0 × 10^5^ cells/mL for 4 h. The phagocytosis efficacy was detected by plate counting.

### Fungal plating

For the mouse colon, dissected tissues were sliced into pieces and homogenized. For cellular culturing, fungal strains were collected by centrifugation at 3000 g for 10 min and inoculated with cells for 4 h in DMEM at a multiplicity of infection (MOI) ratio of 3:1 (fungi : cells). The final density of *C. albicans* varied depending on the inoculum used. By proper dilutions, an aliquot of 100 μL of fungal suspension or tissue homogenates was smeared on YPD agar plates containing 1% penicillin-streptomycin and cultivated for 24–48 h at 37°C. Fungal growth was recorded as colony forming unit (CFU) per millilitre or grams.

### ROS

RAW264.7 cells (3.0 × 10^5^ cells/mL) were seeded for 24 h and treated with 5 mm N-acetylcysteine (NAC, an antioxidant; SparkJade, Jinan, China) for 2 h. After removing NAC, the cells were infected with *C. albicans* at a final concentration of 9.0 × 10^5^ CFU/mL for 4 h. ROS fluorescence was detected using fluorescence microscope (Olympus IX81, Japan) with a Reactive Oxygen Species Assay Kit according to the manufacturer’s instructions (Beyotime, Shanghai, China).

### Apoptosis

RAW264.7 cells (3.0 × 10^5^ cells/mL) were seeded in a plate for 24 h and processed with 50 μM z-VAD-FMK (a caspase inhibitor; SparkJade, Jinan, China) for 1 h. After removing z-VAD-FMK, the cells were infected with *C. albicans* at a final concentration of 9.0 × 10^5^ CFU/mL for 4 h. After centrifugation, the cells were processed with an Annexin V-FITC Apoptosis Detection Kit in the dark at room temperature according to the manufacturer’s instructions (Beyotime, Shanghai, China). The apoptosis rate was monitored and analysed using flow cytometry (BD FACS Celesta, USA) and FlowJo 10.0.

### Immunoblot

The experimental procedures were reported in our previous study with less modifications [25]. The primary antibodies including anti-occludin (1:1000, Biodragon, RM4965), anti-claudin1 (1:1000, Abcam, ab307692), anti-dectin-1 (1:1000, BIOSS, bs-2455 R), anti-phospho-syk (1:800, Zenbio 310,087), anti-syk (1:800, Zenbio 821,293), anti-NF-κB (1:1000, Zenbio, R25149), β-actin antibody (1:5000, Servicebio, GB15003) were incubated with the blotted samples overnight at 4°C. Subsequently, the samples were treated with the secondary antibody anti-mouse IgG (1:10000, Zenbio 511,103) or anti-rabbit IgG (1:10000, Zenbio 511,203) for 1 h at room temperature with gentle agitation on an orbital shaker. The target protein bands were quantified and processed using a Tanon 5200 device (Shanghai, China) and ImageJ 2.1.0.

### Reverse transcription quantitative real-time PCR (RT-qPCR)

The experimental procedures were reported in our previous study with less modifications [25]. Primers used in this study were synthesized by Sangon (Shanghai, China; Table S1). All gene expressions were calculated using the 2^−ΔΔCt^ method and normalized to those of β-actin, U6, and GAPDH as indicated.

### Immunofluorescence staining

The RAW264.7 macrophages were fixed at room temperature in 4% paraformaldehyde for 15 min and washed with three times of PBS for 10 min each time. The cells were then incubated with 50 μL 0.5% TritonX-100 for 10 min. After three washes with PBS, the cells were sealed with goat serum for 1 h, and treated with CD206 mouse monoclonal antibody (1:400, Proteintech 60,143–1-lg) overnight at 4 °C. The cells were then incubated with CoraLite488-linked AffiniPure goat anti-mouse IgG (H+L) (1:500, Proteintech, SA00013–1) for 90 min after washing with PBS. Finally, the fluorescence was observed using a fluorescence microscope (Olympus IX81, Tokyo, Japan).

### Flow cytometry

RAW264.7 cells were collected and incubated with FITC coupled anti-mouse CD206 antibody (E-AB-F1135C, 1:200, Elabscience, Wuhan, China) at room temperature for 15 min. After two washes with sterile PBS, the specimens were processed by flow cytometry (BD FACS Celesta, USA) using the FITC-A channel. A total of 10,000 cells from each group were chosen for examination. Cells that were not incubated with antibodies were used as the negative control. The flow cytometry data were processed using FlowJO 10.0.

### Statistical analysis

All data were analysed using SPSS 23.0 (SPSS Inc, Chicago, IL, USA) and expressed as mean ± standard deviation (SD). All samples were tested for normality using the Shapiro-Wilk test and homogeneity of variance using Levene’s test. For comparisons between two groups, a Student’s t-test was performed, while for multiple group comparisons, a one-way analysis of variance (ANOVA) was carried out. Spearman analysis was performed for correlation analysis. The significance was set as *p* < 0.05.

## Results

### Candida albicans causes intestinal mycobiota dysbiosis

**Figure 1.**
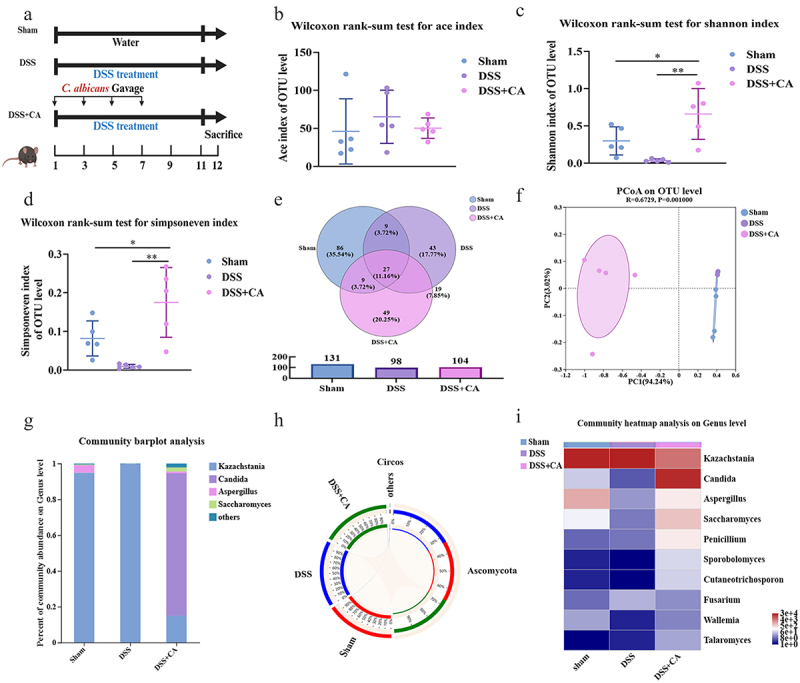
*Candida albicans* causes intestinal mycobiota dysbiosis. (a) Workflow of DSS induced colitis establishment (*n* = 5). (b) ACE index. (c) Shannon index. (d) Simpsoneven index. (e) Venn diagram based on identified OTUs. (f) PCoA analysis based on weighted UniFrac distance. (g) Relative abundance of dominant fungi phyla. (h) Distribution of microbial communities at the phylum level for each sample. Data is visualized by Circos. The bar width of each phylum represents the relative abundance of that phylum in the sample. (i) Community heatmap analysis at the genus level. b was analysed by ANOVA using the Tukey’s test. c and d were analysed by ANOVA using Dunn’s post-hoc test. Data are shown as the mean ± SD. *p < 0.05; **p < 0.01; ***p < 0.001.

Since ITS regions do not belong to the conserved transcribed regions of the structural ribosomal RNAs, ITS sequencing becomes the most common approach to classify fungi [36]. In contrast to the human gut, the mouse gut is naturally free of *C. albicans*, so we firstly evaluated the impact of exogenous *C. albicans* on the intestinal mycobiota by using ITS sequencing (Figure 1(a)). There were no differences in the ACE index among the three groups (Figure 1(b)). Compared with the sham and DSS groups, however, the supplemented *C. albicans* had a great impact on the Shannon and Simpson indices (Figure 1(c,d)). Compared with the sham group (131 OTUs), the supplemented DSS and DSS plus *C. albicans* seemed to decrease the microbial species (98 and 104 OUTs, Figure 1(e)). Using weighted UniFrac distances, the PCoA profiles showed that the microbial structures in the mice co-treated with DSS and CA were significantly altered compared to those in the sham and DSS-treated mice (Figure 1(f)). Apparently, *C. albicans* with interval gavage became the dominant species in DSS-induced colitis instead of *Kazachstasia* (Figure 1(g-i)). These results indicated that *C. albicans* could colonize the mouse gut impaired by DSS and induce intestinal mycobiota dysbiosis.

### MiR-32-5p is a therapeutic target in DSS induced Candida colitis

**Figure 2.**
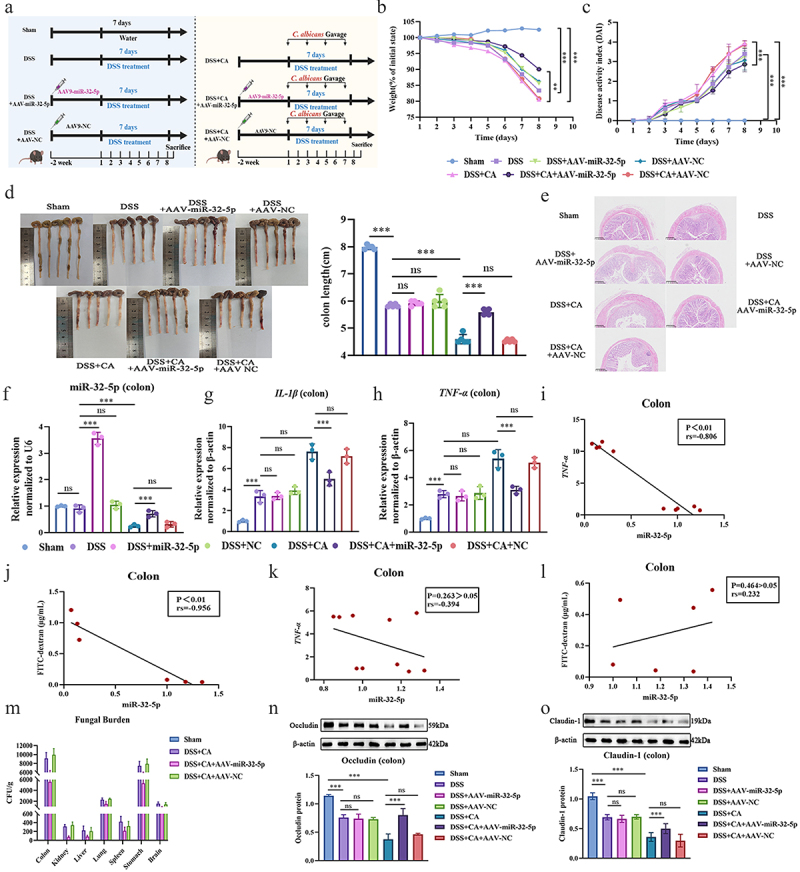
Increased miR-32-5p is protective against DSS induced colitis with *C. albicans* supplementation. (a) Workflow of colitis establishment in mice. The mice are intraperitoneally injected with miR-32-5p adenovirus followed by being normally given water and food at liberty for 2 weeks before DSS gavage. b. Weight loss (*n* = 5). c. Disease activity index (*n* = 5). d. Colon length (*n* = 5). e. Histopathologic changes of colon tissue (*n* = 3). Scale bar: 50 μm. f-h. Relative gene expressions of miR-32-5p, IL-1β and TNF-α in colon tissues (*n* = 3). i-l. Correlations of colon inflammation and intestinal barrier integrity with miR-32-5p in the presence (i, j) or absence (k, l) of *C. albicans* (*n* = 5). i, k. TNF-α versus miR-32-5p (*p* < 0.01,rs = -0.806; *p* = 0.263 > 0.01, rs = -0.394), j, l. FITC-dextran versus miR-32-5p (*p* < 0.01,rs=-0.956; *p* = 0.464 > 0.05, rs = 0.232). (m). Fungal burdens of colon, kidney, liver, lung, spleen, stomach and brain in the presence of *C. albicans* (*n* = 3). n, o. Immunoblots of tight-junction protein. (n). Occludin, (o). Claudin-1. Each experiment was repeated three times. Data are shown as the mean ± SD. B,C,D,F-H,N,O were analysed by one-way analysis of variance using Least Significant Difference (LSD) post hoc test. I-L were analysed by Spearman analysis. *p < 0.05, **p < 0.01, ***p < 0.001, ns: no significance.

Previously, we found that miR-32-5p was the most differentially expressed miRNA and was downregulated nearly 11-fold in the inflamed mouse intestine induced by DSS and *C. albicans* [25]. In this study, we used adenovirus to upregulate miR-32-5p in a DSS-induced colitis model supplemented with *C. albicans* (Figure 2(a)). The results showed that upregulated miR-32-5p can decrease weight loss, DAI score, colon shortening, pathological changes, and inflammatory IL-1β and TNF-α levels (Figure 2(b–h)). We also found that miR-32-5p was negatively correlated with TNF-α (*p* < 0.01, rs = −0.806, Figure 2(i)) and FITC-dextran (*p* < 0.01, rs = −0.956, Figure 2(j)). Interestingly, miR-32-5p appeared to have no therapeutic effects in colitis mice in the absence of *C. albicans* (Figure 2(b–l)). Compared with colitis mice in the absence of *C. albicans*, microbiological inspection revealed that increased miR-32-5p expression by adenovirus transfection could effectively inhibit fungal growth in the intestine and other organs (Figure 2(m), Figure S2A), and the impaired tight-junction proteins occludin and claudin-1 could also be recovered (Figure 2(n,o), Figure S8-S1). We used the normal human colonic mucosal epithelial cell strain NCM460 to further investigate the therapeutic effects of miR-32-5p after co-treatment with DSS and *C. albicans*. In accordance with the animal experiments, the cellular tests also showed that upregulated miR-32-5p could rectify the abnormalities of inflammatory IL-1β and TNF-α levels (Figure S1A-S1C), tight-junction protein occluding and claudin-1 expressions (Figure S1D, S1E and S8-1A-D), LDH level (Figure S1F), and fungal capacity (Figure S1G and Figure S2B) in the presence of *C. albicans*, but seemed have no therapeutic potential in the absence of *C. albicans* (Figure S1A-S1F). Using several intestinal and macrophage cell lines, we noticed that miR-32-5p was markedly downregulated after infections with multiple *C. albicans* strains (Figure S3A-S3J). These results suggest that miR-32-5p may be a universal therapeutic target for *Candida* colitis.

### Fungal β-glucan instructs miR-32-5p to ameliorate Candida colitis through dual inflammatory responses

**Figure 3.**
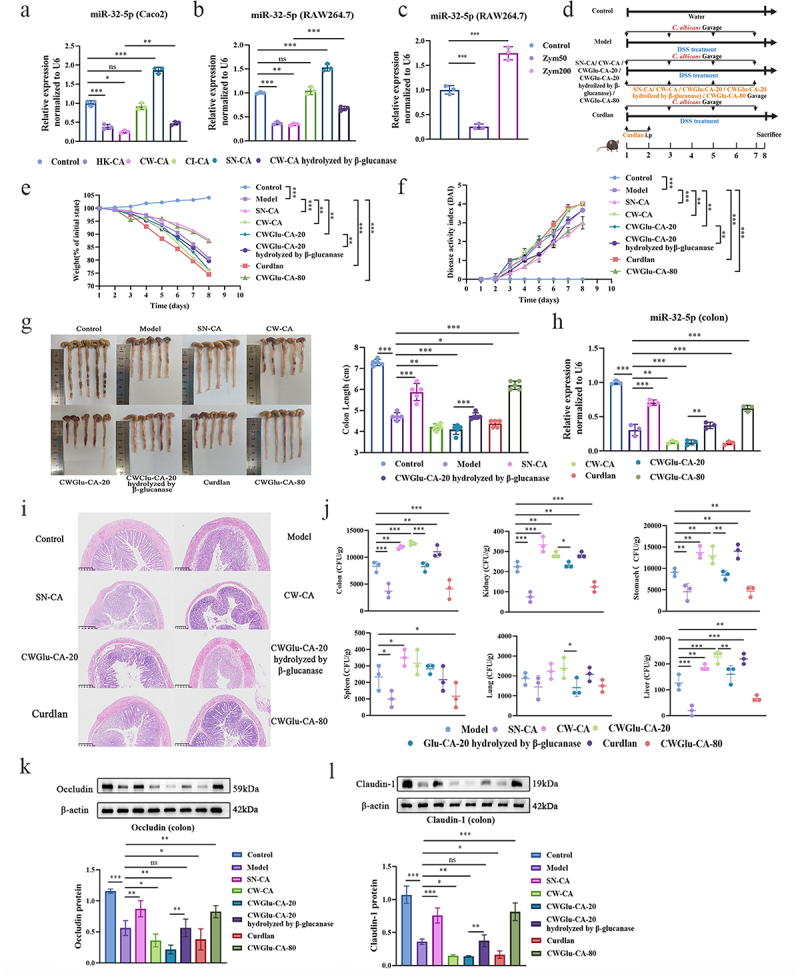
Cell wall β-glucan is the major component to stimulate pro- and anti-inflammatory dual responses through miR-32-5p in *Candida* colitis. a,b. Relative expression of miR-32-5p in (a) Caco2 and (b) RAW264.7 cells (both at 3 × 10^5^ cells/mL) treated with heat-killed *C. albicans*(HK-CA, 9 × 10^5^CFU), cell wall of *C. albicans*(CW-CA, 100 μg/mL), cellular inclusion of *C. albicans*(CI-CA, 9 × 10^5^CFU), supernatant from *C. albicans*at 9 × 10^5^CFU/mL (SN-CA, 1 mL), CW-CA (100 μg/mL) hydrolysed by β-glucanase (0.1 mg/mL) for 4 h. c. Relative expression of miR-32-5p in RAW264.7 cells (3 × 10^5^ cells/mL) treated, respectively, with zymosan at 50 (Zym50) and 200 μg/mL (Zym200) for 4 h. d. Workflow of colitis establishment in the presence of *C. albicans*. The mice were treated with SN-CA (200 μL per mouse from 1 × 10^8^CFU), CW-CA (50 mg/kg), cell wall β-glucan of *C. albicans*at 20 mg/kg (CWGlu-CA-20), CWGlu-CA-20 hydrolysed by β-glucanase (4 mg/mL), curdlan (25 mg/kg), cell wall β-glucan of *C. albicans*at 80 mg/kg (CWGlu-CA-80) on the day 1, 3, 5, 7. e. Weight loss (*n* = 5). f. Disease activity index (*n* = 5). g. Colon length (*n* = 5). h. Histopathologic changes of colon tissue (*n* = 3). Scale bar: 50 μm. i. Relative expression of miR-32-5p in colon tissues (*n* = 3). j. Fungal burdens of colon, kidney, stomach, spleen, lung, and liver (*n* = 3). k,l. Immunoblots of tight-junction protein Occludin and Claudin-1. Each experiment was repeated three times. Data are shown as the mean ± SD. A-C, E-H and J-L were analysed by one-way analysis of variance using the Least Significant Difference (LSD) post hoc test. *p < 0.05, **p < 0.01, ***p < 0.001, ns: no significance.

As *C. albicans* is a potential aetiological agent that modulates miR-32-5p in this model, it is necessary to specify which component(s) of *C. albicans* is responsible for miR-32-5p downregulation. After treatment with heat-killed *C. albicans* (HK-CA), cell wall of *C. albicans* (CW-CA), cellular inclusion of *C. albicans* (CI-CA), supernatant of *C. albicans* (SN-CA), and β-glucanase, we observed that cell wall β-glucan was the major fungal component that suppressed miR-32-5p (Figure 3(a,b)). In contrast, SN-CA, which also contained a certain amount of secreted fungal β-glucan, strikingly upregulated miR-32-5p (Figure 3(a,b)). We presumed that fungal β-glucan could generate pro- and anti-inflammatory responses to *Candida* colitis in a concentration-dependent manner, which was verified in RAW264.7 pre-treated with zymosan at 50 (downregulated) and 200 μg/mL (upregulated, Figure 3(c)). Coincidently, both cell wall β-glucan extraction from *C. albicans* at 20 mg/kg (CWGlu-CA-20) and curdlan at 25 mg/kg downregulated miR-32-5p, aggravated *Candida* colitis, promoted fungal growth, and impaired the intestinal mucosal barrier. On the contrary, both cell wall β-glucan extraction from *C. albicans* at 80 mg/kg (CWGlu-CA-80) and SN-CA upregulated miR-32-5p, ameliorated *Candida* colitis, inhibited fungal colonization, and recovered gut mucosal integrity. However, hydrolyzation of β-glucanase abolished the exacerbated effect of Glu-CA-20 on *Candida* colitis (Figure 3(d–i), Figure S4 and S8-S2A-D). Interestingly, β-glucan could instruct the relative expression of miR-32-5p, but had no therapeutic effect on colitis in the absence of *C. albicans* (Figure S5A-S5H and S8-S2).

### Fungal β-glucan hinders miR-32-5p mediated inflammation soaring

**Figure 4.**
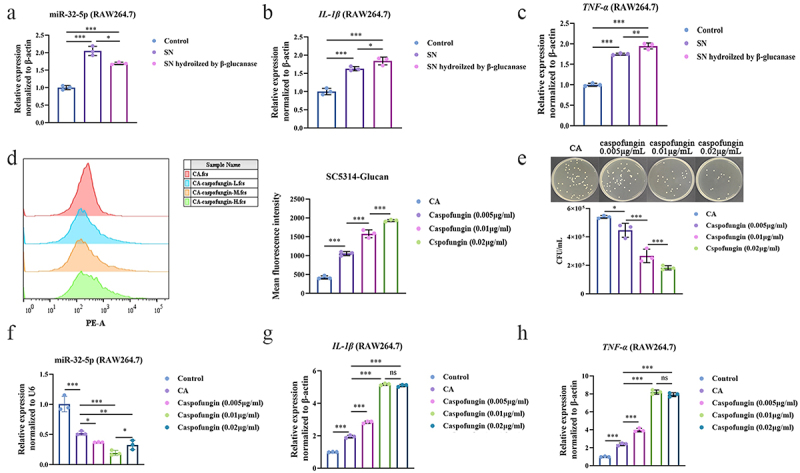
Fungal β-glucan restricts ever-increasing inflammation. (a–c). Relative expressions of miR-32-5p, TNF-α and IL-1β in RAW264.7 cells (3 × 10^5^ cells/mL) treated with isometric supernatant from *C. albicans* at 9 × 10^5^CFU/mL (SN-CA, 1 mL) and/or β-glucanase (0.01 mg/mL) for 4 h. (d). Flow cytometry analysis of exposed cell wall β-glucan of *C. albicans* (1 × 10^6^ CFU/mL) induced by caspofungin at 0.005, 0.01 and 0.02 μg/mL for 24 h. (e). Fungal counting in RAW264.7 (3 × 10^5^ cells/mL) infected with *C. albicans* (9 × 10^5^ CFU/mL) after co-culture with caspofungin at 0.005, 0.01 and 0.02 μg/mL for 24 h. (f-h). Relative expressions of miR-32-5p, TNF-α and IL-1β. The experimental conditions are the same as E. Each experiment was repeated three times. Data are shown as the mean ± SD. (a-h) were analyzed by one-way analysis of variance using Least Significant Difference (LSD) post hoc test. *p < 0.05, **p < 0.01, ***p < 0.001, ns: no significance.

The abovementioned results showed that low fungal β-glucan (CWGlu-CA-20 or Zym50) induced the downregulation of miR-32-5p and the increase of pro-inflammatory cytokines followed by aggravated colitis severity, while high fungal β-glucan (CWGlu-CA-50 or Zym200) induced the upregulation of miR-32-5p and the decrease of pro-inflammatory cytokines accompanied by relieved *Candida* colitis (Figure 2 and 3). Paradoxically, these results imply an “anti-inflammatory” effect of high fungal β-glucan in *Candida* colitis. During maturation and dispersion, a part of *C. albicans* β-glucan settles on the fungal cell wall, while the remnants are transported across the cell wall and secreted into the supernatant [37]. After enzymolysis by glucanase, upregulated miR-32-5p in conjunction with decreased TNF-α and IL-1β caused by SN-CA was abolished compared with free enzymolysis (Figure 4(a–c)). However, the relative expression levels of TNF-α and IL-1β were still higher than those free of treatments (Figure 4(a–c)), indicating an inflammatory context after enzymolysis. Caspofungin can induce exposure of the fungal cell wall β-glucan and enhance the phagocytosis of fungi [38]. Here, concentration-dependent intensity of β-glucan exposure induced by caspofungin was demonstrated (Figure 4(d)). In agreement, the inhibitory effect on fungal growth was also dependent on caspofungin concentration (Figure 4(e)). In contrast to caspofungin at 0.005 and 0.01 μg/mL that significantly downregulated miR-32-5p, caspofungin at 0.02 μg/mL could apparently upregulate miR-32-5p and was hard to increase TNF-α and IL-1β compared with 0.01 μg/mL (Figure 4(f–h)). In terms of more β-glucan exposure induced by caspofungin at 0.02 μg/mL relative to 0.01 μg/mL, the results suggest that fungal β-glucan can keep ever-increasing inflammation under control through miR-32-5p in a concentration-dependent manner.

### MiR-32-5p requires Dectin-1 signaling mediated ROS to eliminate fungi

**Figure 5.**
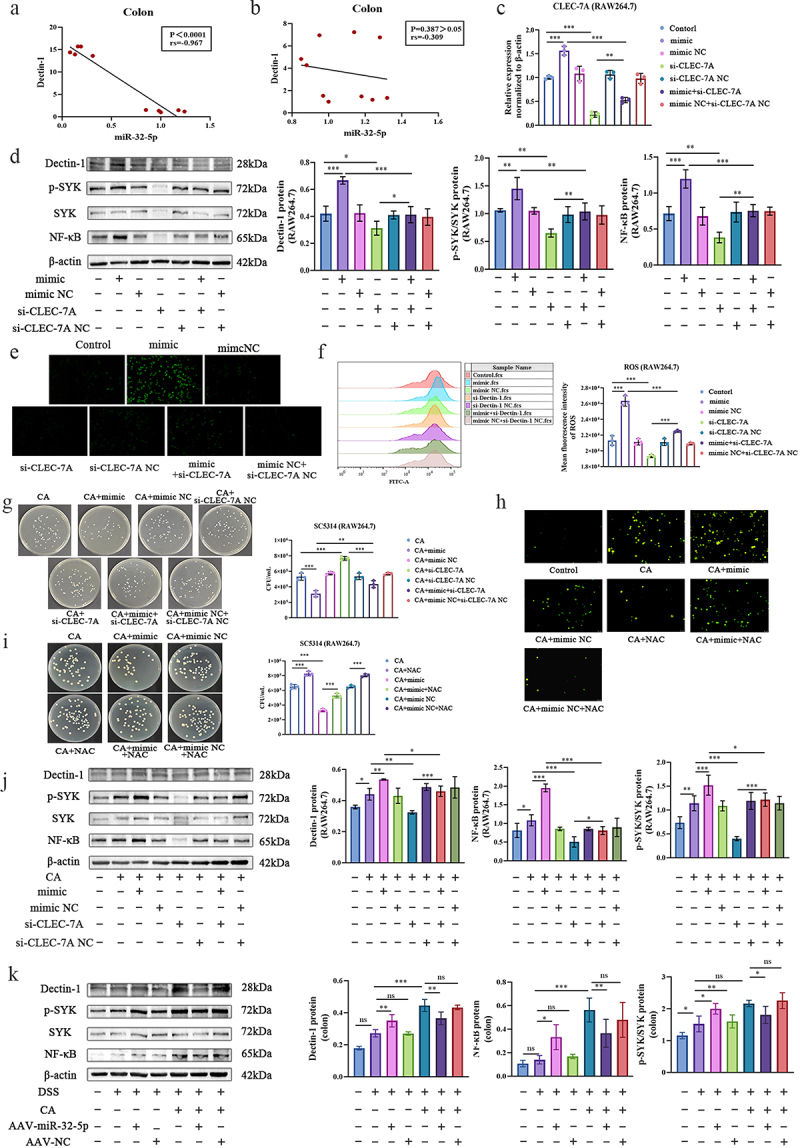
MiR-32-5p facilitate fungal removal through Dectin-1 mediated ROS.

Since *C. albicans* is the major driving force that causes microbiota and mycobiota dysbiosis and aggravates DSS-induced colitis (Figures 1 and 2) [9], the elimination of *C. albicans* is the first priority in restoring intestinal flora homoeostasis and relieving colitis severity. Since Dectin-1 is the major receptor to recognize fungal β-glucan and can activate antifungal innate immune response by enhancing phagocytosis and ROS [39], we are interested to explore whether miR-32-5p mediated fungal elimination is associated with Dectin-1 signalling and ROS in macrophage. In *Candida* colitis model, we demonstrated in this study and elsewhere that there was a strong negative correlation between miR-32-5p and Dectin-1 (Figure 5(a)) [25], and this relationship disappeared in colitis free of *C. albicans* gavage (Figure 5(b)). However, upregulated miR-32-5p led to increased Dectin-1 signalling in RAW264.7 cells (Figure 5(c,d)) and enhanced fungistatic potential in *Candida* colitis mice (Figure 3(j)) and Caco2 cells [25]. These results were partly consistent with the effects of high fungal β-glucan (Figure 3(c,h,j)). Restriction of intestinal *C. albicans* overgrowth was verified to be a driving force to alleviate colitis severity in this study (Figure 3(e–g), (i) ,(k-l)). As expected, miR-32-5p mimic significantly increased CLEC-7A expression (Figure 5C), activated Dectin-1 associated signalling (Figure 5(d)), and promoted ROS production (Figure 5(e,f)). These alterations were largely abolished by transfection with si-Dectin-1 (Figure 5(c–f), Figure S6 and S8-S3A-C). In the presence of *C. albicans*, increased miR-32-5p markedly inhibited fungal load by activating Dectin-1 associated signalling, and silencing CLEC-7A and suppression of ROS abrogated the antifungal effect of upregulated miR-32-5p (Figure 5(g–j) and Figure S8-S3D-F). Intriguingly, overexpressed miR-32-5p could activate Dectin-1 signalling in the colitis mice free of *C. albicans* and inhibit Dectin-1 signalling in the *Candida* colitis mice which might be mainly due to the removal of *C. albicans* (Figure 5(k) and Figure S8-S3G-I). These results suggest that miR-32-5p participates in fungal elimination through Dectin-1 signalling associated ROS.

(a,b). Correlations of Dectin-1 with miR-32-5p in colitis gut (a) in the presence of *C. albicans*(*p* < 0.0001, rs = -0.967) and (b) in the absence of *C. albicans*(*p* = 0.387 > 0.05, rs = -0.309). c. Relative expression of CLEC-7A in RAW264.7 after treatment of miR-32-5p mimic and/or si-CLEC-7A. d. Immunoblots and quantifications of Dectin-1 signalling in RAW264.7 after treatment of miR-32-5p mimic and/or si-CLEC-7A. e. Representative fluorescent photos of ROS in RAW264.7 after treatment of miR-32-5p mimic and/or si-CLEC-7A. Magnification: ×200. f. Flow cytometry analysis of ROS in RAW264.7 after treatment of miR-32-5p mimic and/or si-CLEC-7A. g. Fungal plate counting after treatment of miR-32-5p mimic and/or si-CLEC-7A in RAW264.7. h. Representative fluorescent photos of ROS in the presence of *C. albicans*after treatment of miR-32-5p mimic and/or NAC in RAW264.7. Magnification: ×200. i. Fungal plate counting after treatment of miR-32-5p mimic and/or N-acetylcysteine (NAC) in RAW264.7. j. Immunoblots and quantifications of Dectin-1 signalling in the presence of *C. albicans*after treatment of miR-32-5p mimic and/or si-CLEC-7A in RAW264.7. k. Immunoblots of Dectin-1 signalling in colitis colon tissues in the presence or absence of *C. albicans* after intraperitoneal injection of miR-32-5p adenovirus. Each experiment was repeated three times. Data are shown as the mean ± SD. a and b were analysed using Spearman analysis. c,d,f,g,i-k were analysed by one-way analysis of variance using the Least Significant Difference (LSD) post hoc test. *p < 0.05, **p < 0.01, ***p < 0.001.

### MiR-32-5p modulates macrophage apoptosis independent of Dectin-1 receptor

**Figure 6.**
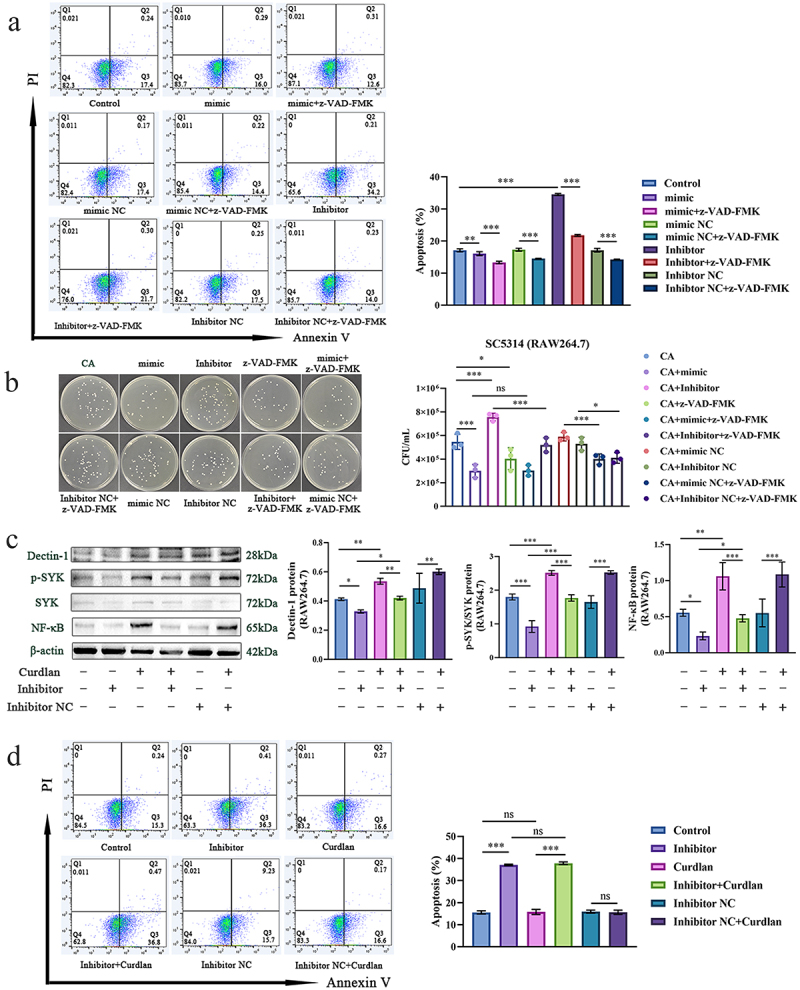
miR-32-5p influences macrophage apoptosis in a Dectin-1 receptor independent manner.

Since the elevation of ROS is inclined to induce cellular apoptosis, we intended to determine whether miR-32-5p could modulate the apoptosis of macrophage through Dectin-1. The results showed that downregulated miR-32-5p could remarkably promote apoptosis and inhibit fungal growth in RAW264.7 cells (Figure 6(a,b) and Figure S7A). These effects were abolished in the presence of the pan-caspase inhibitor z-VAD-FMK (Figure 6(b)). z-VAD-FMK can irreversibly bind to the catalytic sites of caspase 3/7/8, thereby specifically inhibiting apoptosis. Although upregulated miR-32-5p could significantly inhibit apoptosis of RAW264.7, the elevated miR-32-5p was unexpectedly ineffective against fungal proliferation after treatment with z-VAD-FMK (Figure 6(b)). These observations suggest that increased apoptosis of macrophage impairs the innate antifungal response to *C. albcians*. Subsequently, we aimed to investigate whether the Dectin-1 receptor and associated signalling were involved in miR-32-5p mediated macrophage apoptosis by using miR-32-5p inhibitor. In contrast to miR-32-5p mimic (Figure 5(d)), miR-32-5p inhibitor could strikingly lessen Dectin-1 (Figure 6(c)). Although the Dectin-1 agonist effectively activated Dectin-1 signalling (Figure 6(c), Figure S8-4A-C), curdlan seemed to have negligible impact on macrophage apoptosis when used in combination with miR-32-5p inhibitor as compared with miR-32-5p inhibitor used alone (Figure 6(d) and S7B). These results imply that miR-32-5p could modulate the apoptosis-mediated antifungal function of macrophages in a Dectin-1 independent manner.

(a). Flow cytometry analysis of apoptosis in RAW264.7 stained by PI and Annexin V in the presence of miR-32-5p mimic/inhibitor and/or z-VAD-FMK (b). Fungal plating in the presence of miR-32-5p mimic/inhibitor and/or z-VAD-FMK (c). Immunoblots of Dectin-1 signalling in RAW264.7 in the presence of miR-32-5p inhibitor and/or curdlan (1 mg/mL) (d). Flow cytometry analysis of apoptosis in RAW264.7 stained by PI and Annexin V in the presence of miR-32-5p inhibitor and/or curdlan (1 mg/mL) Each experiment was repeated three times. Data are shown as the mean ± SD. (a-d) were analysed by one-way analysis of variance using the Least Significant Difference (LSD) post hoc test. *p < 0.05, **p < 0.01, ***p < 0.001, ns: no significance.

### MiR-32-5p polarizes M2a phenotype of macrophage

**Figure 7.**
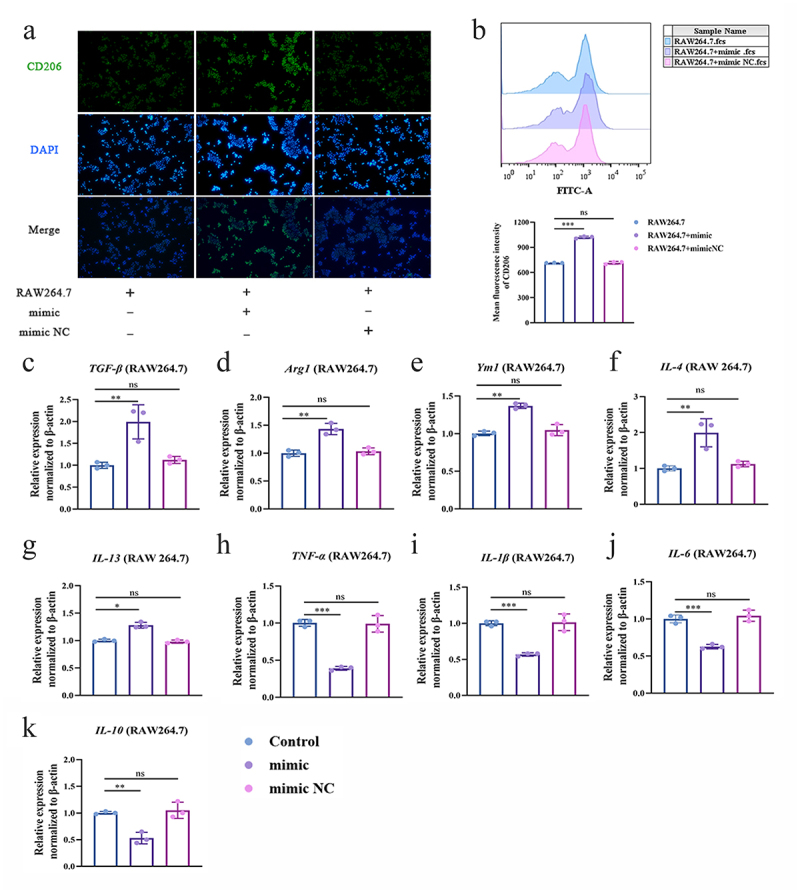
MiR-32-5p induces polarization of M2a phenotype. a. Representative fluorescent photos of CD206 expression stained by DAPI and anti-CD206 in RAW264.7 treated with miR-32-5p mimic. Magnification: ×200.b. Flow cytometry analysis of CD206 in RAW264.7 treated with miR-32-5p mimic.c-k. Relative expressions of a panel of M2a phenotype genes. (c) TGF-β, (d) Arg1, (e) Ym1, (f) IL-4, (g) IL-13, (h) TNF-α, (i) IL-1β, (j) IL-6, (k) IL-10. Each experiment was repeated three times. Data are shown as the mean ± SD. B-K were analysed by one-way analysis of variance using the Least Significant Difference (LSD) post hoc test. *p < 0.05, **p < 0.01, ***p < 0.001, ns: no significance.

We found that upregulated miR-32-5p decreased the relative expressions of pro-inflammatory cytokines TNF-α and IL-1β in NCM460 (Figure S1A and S1B) and Caco2 cells [25]. Moreover, the miR-32-5p mimic significantly enhanced the relative expression of CLEC-7A (Figure 5(c)), which is an important hallmark of the polarization of the M2a phenotype [40]. Therefore, we monitored a panel of representative genes of the M2a phenotype. The results showed that increased miR-32-5p conspicuously induced CD206 expression (Figure 7(a,b)), increased the relative expressions of TGF-β, Arg-1, Ym1, IL-4, and IL-13 (Figure 7(c-g), and decreased the expression of TNF-α, IL-1β, and IL-6 (Figure 7(h-j)) in RAW264.7 cells. Surprisingly, IL-10 was inhibited in the presence of miR-32-5p mimic (Figure 7(k)). These results imply that miR-32-5p regulates the polarization of M2a macrophages.

## Discussion

Fungal β-glucan, a pivotal pathogen associated molecular pattern (PAMP), is usually buried underneath the fungal cell wall and can be transported across the cell wall and secreted into the extracellular context [37]. An increasing number of reports support the use of serum β-glucan as an indicator for therapeutically monitoring *Clostridium difficile*-associated diarrhoea and IBD [41–43]. Under normal condition, budding yeast and cell separation generate permanent scars that expose sufficient β-glucan to initiate antifungal responses [13,44]. Innate cells can also unmask *Candida* cell wall β-glucan and augment innate immune recognition during disseminated candidiasis [45]. However, several reports have demonstrated that fungal β-glucan can exert both protective and destructive effects in colitis [46–48]. Consistently, we found in this study that the extracted *Candida* cell wall β-glucan had dual effects on *Candida* colitis in a concentration-dependent manner through intestinal miR-32-5p. Interestingly, we noticed that the soluble β-glucan in the supernatant appeared to control the overloaded pro-inflammation by up-regulating intestinal miR-32-5p. Paradoxically, elevated serum β-glucan levels are tightly correlated with invasive fungal infections in patients with impaired immunity in physiological status. The lower the host immunity, the higher the serum fungal β-glucan level [49]. In fact, it seems that soluble β-glucan exerts anti-inflammatory effects contrary to pro-inflammatory reactions induced by exposed β-glucan [50,51]. Furthermore, Gonzalez et al. reported an inverse correlation between serum glucan and serum cytokine levels in ICU patients with infections when serum glucan concentrations were low. They concluded that circulating glucans might restrict the cytokine increase in response to infection [52].

Most documents relevant to miR-32-5p focus on the investigation of cancer. No defined effect or mechanism of miR-32-5p has been explored in UC [53]. Previously, miR-32-5p was reported to be up-regulated in *Mycobacterium tuberculosis*-infected macrophages and *Helicobacter pylori*-induced paediatric enteritis [54,55]. We identified miR-32-5p as the most differentially expressed miRNA in DSS-induced colitis following *C. albicans* supplementation. Compared to colitis free of *C. albicans*, miR-32-5p was markedly downregulated in the presence of *C. albicans* [25]. In this study, we found that intestinal miR-32-5p expression was negatively correlated with the Dectin-1 receptor, TNF-α expression and gut mucosal barrier integrity in *Candida* colitis, and the down-regulation of miR-32-5p is a universal result caused by *C. albicans* in multiple cells. We further uncovered that alterations in intestinal miR-32-5p were governed by fungal β-glucan in a dose-dependent manner. Increased miR-32-5p level could effectively inhibit fungal growth and reduce colitis severity. As a result, we identified miR-32-5p as a characteristic potential target in *Candida* colitis.

M1 macrophages are normally linked to the onset of IBD, and anti-TNF-α therapy can ameliorate the disease by increasing the M2 phenotype [56–58]. M2a macrophages are principally induced by IL-4 and IL-13 and can markedly produce a signature of markers and cytokines including IL-10, TGF-β, CD206, Ym1 (Chil3) and Arg1 [22]. Generally, M2a macrophages have low levels of pro-inflammatory cytokines, such as TNF-α, IL-1β and IL-6. These alterations were consistent with the majority of our results. Intriguingly, a genome-wide M1/M2a transcriptional comparison study showed that the Dectin-1 receptor was remarkably induced by IL-4 [40]. Coincidentally, PPARγ ligands reportedly instructed a phenotypic M2b-to-M2a switch in peritoneal macrophages, which also expressed high levels of Dectin-1, favouring the elimination of gastrointestinal *C. albicans* [59]. It has been discovered that Dectin-1 could directly trigger phagocytosis via Syk, which contributed to fungicidal function, but had an indirect effect on TLR-mediated inflammatory cytokines [39,60]. As shown previously, sulfasalazine-induced M2a polarization enhanced phagocytosis and fungal clearance in an IL-4 Rα-independent manner [61]. Moreover, M2a macrophages are conductive to the resolution of DSS-induced colitis in TLR4-SNP mice [62]. In this study, miR-32-5p stimulated Dectin-1 expression and inhibited fungal intestinal growth by modulating cytokine production. Interestingly, we observed that miR-32-5p increased IL-4 and IL-13 levels in the absence of *C. albicans*. Kiyonao et al. observed that β-glucan (curdlan) could activate Dectin-1/Syk signalling to stimulate secretion of IL-4 and IL-13 which were assumed to induce macrophage migration into inflammation sites followed by antifungal immunity [63]. In the present scenario, we suppose that miR-32-5p can elevate IL-4 and IL-13 levels, which readily facilitate M2a polarization, thereby promoting an antifungal response to *C. albicans*.

Incremental documents have shown that elevated intracellular ROS and apoptosis of macrophages are two hallmarks of the anti-*Candida* process [64,65]. However, our results provide a new anti-*Candida* strategy for M2a through Dectin-1 mediated ROS enhancement. As demonstrated previously, Dectin-1 directly stimulated the production of ROS after engagement with β-glucan, facilitating fungal killing [39]. Although macrophage polarization can’t be specifically ascribed to the metabolic status, M2 macrophages are generally correlated with increased oxidative phosphorylation and fatty acid oxidation, which are associated with ROS production [66]. Our results further reveal that miR-32-5p-induced ROS production is concomitant with apoptosis reduction. Coincidentally, Tomita et al. demonstrated that apoptosis inhibitors in macrophages could alleviate fungus-induced peritoneal injury [67]. A recent study by Jiang et al. further showed that apoptosis could enhance *C. albicans* infections via AKT signalling in macrophages [68]. Despite that miR-32-5p mediated macrophage ROS production and apoptosis are responsible for intestinal anti-*C. albicans* and colitis amelioration, the requirements of both processes for Dectin-1 associated signalling are fundamentally different, which warrants in-depth exploration in the following study.

**Figure 8.**
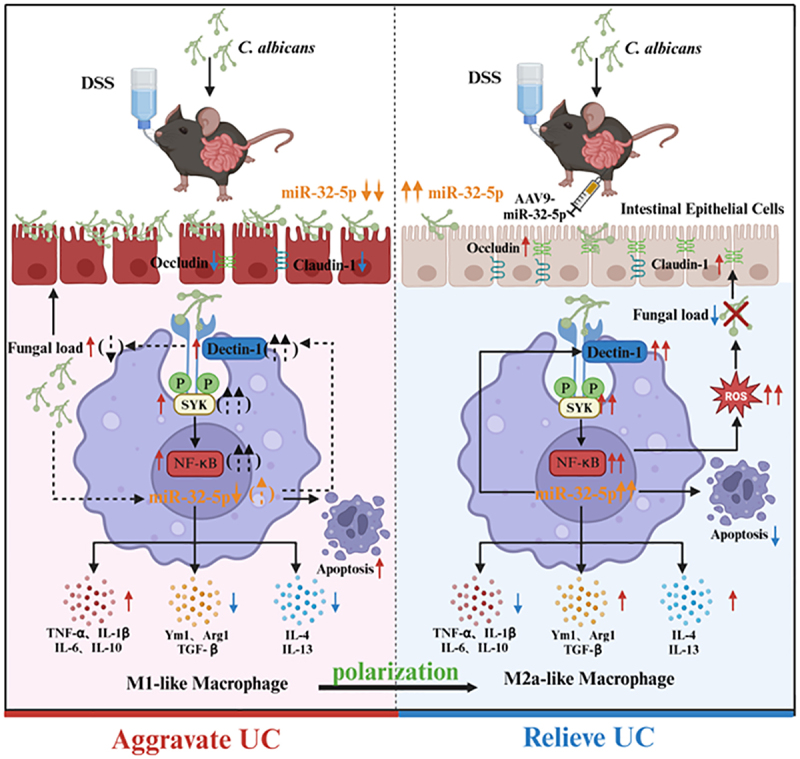
Schematic workflow of this study.

Collectively, we come up with a novel therapeutic mechanism of miR-32-5p against *Candida* colitis in this study (Figure 8). We propose that the potential of miR-32-5p to induce macrophage polarization of the M2a phenotype might be a universal strategy for the treatment of *Candida* associated infections.

## Supplementary Material

Clean copy of supplementary figure legends and supplementary table.docx

Figure S3.tif

Figure S4.tif

Figure S7.tif

Figure S5.tif

Figure S8 4.tif

Figure S1.tif

Figure S8 3.tif

Figure S8 1.tif

Figure S6.tif

ARRIVE guidelines.pdf

Figure S2 - QVIR-2024-0854.R1.tif

Figure S8 2.tif

## Data Availability

The data that support the findings of this study are openly available in Science Data Bank at http://doi.org/10.57760/sciencedb.19631.
