# Supplementary material for: Fungal β-glucan instructed miR-32-5p modulates Dectin-1 signaling mediated inflammation, reactive oxygen species and apoptosis through polarization of “M2a-like” macrophage in Candida colitis
Source: Virulence. 2025 Jun 6;16(1):2514789. doi: 10.1080/21505594.2025.2514789 (PMC12147485; doi:10.1080/21505594.2025.2514789)
Supplement: Clean copy of supplementary figure legends and supplementary table.docx [file KVIR_A_2514789_SM6655.docx]

**Supplementary Material**

Fungal β-glucan instructed miR-32-5p modulates Dectin-1 signaling mediated inflammation, reactive oxygen species and apoptosis through polarization of “M2a-like” macrophage in *Candida* colitis

Running title: Role of miR-32-5p in UC

Liu Yang ^a,1^, Chengcheng Liu ^a,1^, Hanyu Zhu ^a,1^, Zixu Wang ^a^, Qinai Luo ^b^, Yuzhe Huang^c^, Hao Wang ^d,e,*^, Min Hu ^b,e,^, Jing Shao ^a,e,*^

^a^ Laboratory of Infection and Immunity, College of Integrated Chinese and Western Medicine (College of Life Science), Anhui University of Chinese Medicine, 433 Room, Zhijing Building, 350 Longzihu Road, Xinzhan District, Hefei 230012, Anhui, P. R. China

^b^ Department of pathology, College of Integrated Chinese and Western Medicine (College of Life Science), Anhui University of Chinese Medicine, Zhijing Building, 350 Longzihu Road, Xinzhan District, Hefei 230012, Anhui, P. R. China

^c^ Department of Pharmacy, Anhui University of Chinese Medicine, 350 Longzihu Road, Xinzhan District, Hefei 230012, Anhui, P. R. China

^d^ Anhui Province Key Laboratory of Meridian Viscera Correlationship, Anhui University of Chinese Medicine, Zhijing Building, 350 Longzihu Road, Xinzhan District, Hefei 230012, Anhui, P. R. China

^e^ Institute of Integrated Traditional Chinese and Western Medicine, Anhui Academy of Chinese Medicine, Zhijing Building, 350 Longzihu Road, Xinzhan District, Hefei 230012, Anhui, P. R. China

^1^ These authors contribute equally to this paper.

*Corresponding author: Dr. Jing Shao, E-mail: [ustcnjnusjtu@126.co](mailto:ustcnjnusjtu@126.co)m or [sq@ahtcm.edu.cn](mailto:sq@ahtcm.edu.cn); Dr. Hao Wang, E-mail: haohao19870521@126.com.

**Figure S1.** Effects of miR-32-5p on DSS-treated NCM460 cells in the presence or absence of *C. albicans*.
A-C. Relative gene expressions (n=3). A. TNF-α, B. IL-1β, C. miR-32-5p.
D,E. Immunoblots of tight-junction protein occluding and claudin-1.
F. Lactate dehydrogenase (LDH) level (n=3).
G. Fungal capacity (n=3).
Each experiment is repeated for three times. Data are shown as mean ± SD. A-G were analyzed by one-way analysis of variance using Least Significant Difference (LSD) post hoc test. * p<0.05, ** p<0.01, *** p<0.001, ns: no significance.

**Figure S2.** Effects of miR-32-5p on fungal growth.
A. Plate counting of colonic tissue homogenates from DSS-induced colitis mice in the presence of *C. albicans* after treatment with miR-32-5p adenovirus (corresponding to Figure 2M).
B. Plate counting in NCM460 cells that are induced with DSS and *C. albicans* after treatment with miR-32-5p mimic (corresponding to Figure S1E).

**Figure S3.** MiR-32-5p is universally decreased in multiple cell types and *Candida albicans* strains.
Relative expression of miR-32-5p in (A, H-J) RAW264. 7 infected with *C. albicans* SC5314, Z4935, Z5172, Z5214, and (B) THP1, (C) Caco2, (D) HT29, (E) HCT116, (F) hs-174T, (G) NCM460 infected with *C. albicans* SC5314.
Each experiment is repeated for three times. Data are shown as mean ± SD. A-F and H-J were analyzed by one-way analysis of variance using Least Significant Difference (LSD) post hoc test. G was analyzed by a two-sided student’s t test. * p<0.05, ** p<0.01, *** p<0.001, ns: no significance.

**Figure S4.** Effects of β-glucan on fungal growth in DSS-induced colitis mice with supplementation of *C. albicans* (corresponding to Figure 3J).

**Figure S5.** β-glucan instructed miR-32-5p does not affect colitis severity in the absence of *Candida albicans*.

A. Workflow of colitis establishment in the absence of *C. albicans*.

B. Weight loss (n=5).

C. Disease activity index (n=5).

D. Colon length (n=5).

E. Relative expression of miR-32-5p in colon tissues (n=3).

F. Histopathologic changes of colon tissue (n=3). Scale bar: 50μm.

G,H. Immunoblots of tight-junction protein occluding and claudin-1.

Each experiment is repeated for three times. Data are shown as mean ± SD. B-H were analyzed by one-way analysis of variance using Least Significant Difference (LSD) post hoc test. * p<0.05, ** p<0.01, *** p<0.001, ns: no significance.

**Figure S6.** Silencing efficiency of the three si-Dectin-1 sequences. By PCR, si-CLEC7A-3 was the most effective sequence to inhibit CLEC7A and selected for following experiments.

Each experiment is repeated for three times. Data are shown as mean ± SD and analyzed by one-way analysis of variance using Least Significant Difference (LSD) post hoc test. * p<0.05, ** p<0.01, *** p<0.001, ns: no significance.

**Figure S7.** Fluorescent staining of macrophage apoptosis.

A. Representative fluorescent photos of apoptosis in RAW264.7 stained by PI and Annexin V in the presence of miR-32-5p mimic/inhibitor and/or z-VAD-FMK

B. Representative fluorescent photos of apoptosis in RAW264.7 stained by PI and Annexin V in the presence of miR-32-5p inhibitor and/or curdlan (1mg/mL). Magnification: ×200.

**Figure S8-1.** Uncropped WB gels of (A) Occludin and (B) Claudin-1 in colon tissues corresponding to Figure 2N and 2O, (C) Occludin and (D) Claudin-1 in NCM460 cells corresponding to Figure S1D and S1E.

**Figure S8-2.** Uncropped WB gels of (A) Occludin and (B) Claudin-1 in colitis with *C. albicans* supplementation corresponding to Figure 3K and 3L, (C) Occludin and (D) Claudin-1 in colitis free of *C. albicans* corresponding to Figure S5H and S5I.

**Figure S8-3.** Uncropped WB gels of (A,D,G) Dectin-1, (B,E,H) p-syk and syk, (C,F,I) NF-κB. A-C correspond to Figure 5D. D-F correspond to Figure 5J. G-I correspond to Figure 5K.

**Figure S8-4.** Uncropped WB gels of (A) Dectin-1, (B) p-syk and syk, (C) NF-κB corresponding to Figure 6C.

**Table S1. PCR primers**

| **Target Gene** |  | **Sequence** |
| --- | --- | --- |
| mmu-miR-32-5p | Forward | AAGCGCCTTATTGCACATTACT |
|  | Reverse | CAGTGCAGGGTCCGAGGT |
|  | RT primer | GTCGTATCCAGTGCAGGGTCCGAGGTATTCGCACTGGATACGACTGCAAC |
| U6 | Forward | AGAGAAGATTAGCATGGCCCCTG |
|  | Reverse | ATCCAGTGCAGGGTCCGAGG |
|  | RT primer | GTCGTATCCAGTGCAGGGTCCGAGGTATTCGCACTGGATACGACAAAATA |
| CLCE-7A (for colon tissue and RAW264.7) | Forward | GGGCTGTTTCAGGGTTTGGGTTAG |
|  | Reverse | AGGTAGGCAGAGCACTTGAATGTTC |
| TNF-α (NCM460) | Forward | AGCCCTGGTATGAGCCCATCTATC |
|  | Reverse | TCCCAAAGTAGACCTGCCCAGAC |
| TNF-α (for colon tissue and RAW264.7) | Forward | AAAGGACACCATGAGCACTGAAAG |
|  | Reverse | AGGAGAAGAGGCTGAGGAACAAG |
| IL-1β (for NCM460) | Forward | GCCAGTGAAATGATGGCTTATT |
|  | Reverse | AGGAGCACTTCATCTGTTTAGG |
| IL-1β (for colon tissue and RAW264.7) | Forward | ATGGCTTATTACAGTGGCAATGAGG |
|  | Reverse | AGTGGTGGTCGGAGATTCGTAG |
| IL-4 (for RAW264.7) | Forward | TGAACGAGGTCACAGGAGAA |
|  | Reverse | CGAGCTCACTCTCTGTGGTG |
| IL-13 (for RAW264.7) | Forward | TGTGTCTCTCCCTCTGACCC |
|  | Reverse | CACACTCCATACCATGCTGC |
| TGF-β (for RAW264.7) | Forward | CAGTCAGCTGGCCTTGGTC |
|  | Reverse | CAACTTCTTCTCCCCGCCAT |
| Arg1 (for RAW264.7) | Forward | AGCACTGAGGAAAGCTGGTC |
|  | Reverse | TACGTCTCGCAAGCCAATGT |
| Chil3 (for RAW264.7) | Forward | TCTGGGTACAAGATCCCTGAA |
|  | Reverse | TTTCTCCAGTGTAGCCATCCTT |
| IL-6(for RAW264.7) | Forward | CCACTCCCAACAGACCTGTCTA |
|  | Reverse | CTGCAAGCCAGTTTGGTAGCATC |
| IL-10 (for RAW264.7) | Forward | GGGGCCAGTACAGCCGGGAA |
|  | Reverse | CTGGCTGAAGGCAGTCCGCA |
| GAPDH (for NCM460) | Forward | ATGGAGAAGGCTGGGGCTC |
|  | Reverse | AAGTTGTCATGGATGACCTTG |
| β-actin (for colon tissue and RAW264.7) | Forward | ATCTGGCACCACACCTTCTACAATG |
|  | Reverse | CACGCTCGGTCAGGATCTTCATG |
