# Supplementary figures and images for: Fungal β-glucan instructed miR-32-5p modulates Dectin-1 signaling mediated inflammation, reactive oxygen species and apoptosis through polarization of “M2a-like” macrophage in Candida colitis
Source: Virulence. 2025 Jun 6;16(1):2514789. doi: 10.1080/21505594.2025.2514789 (PMC12147485; doi:10.1080/21505594.2025.2514789)

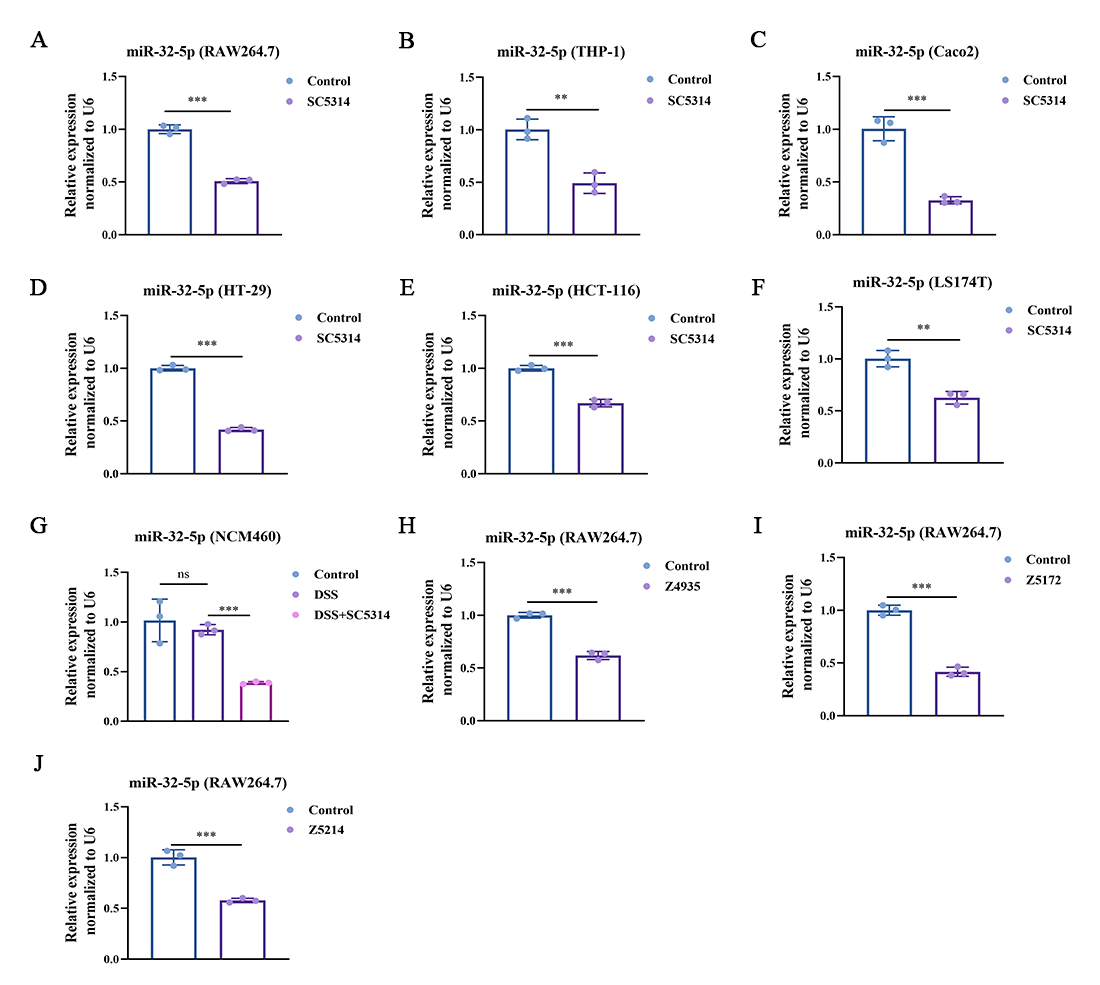

Supplement: Figure S3.tif [file KVIR_A_2514789_SM6654.tif]

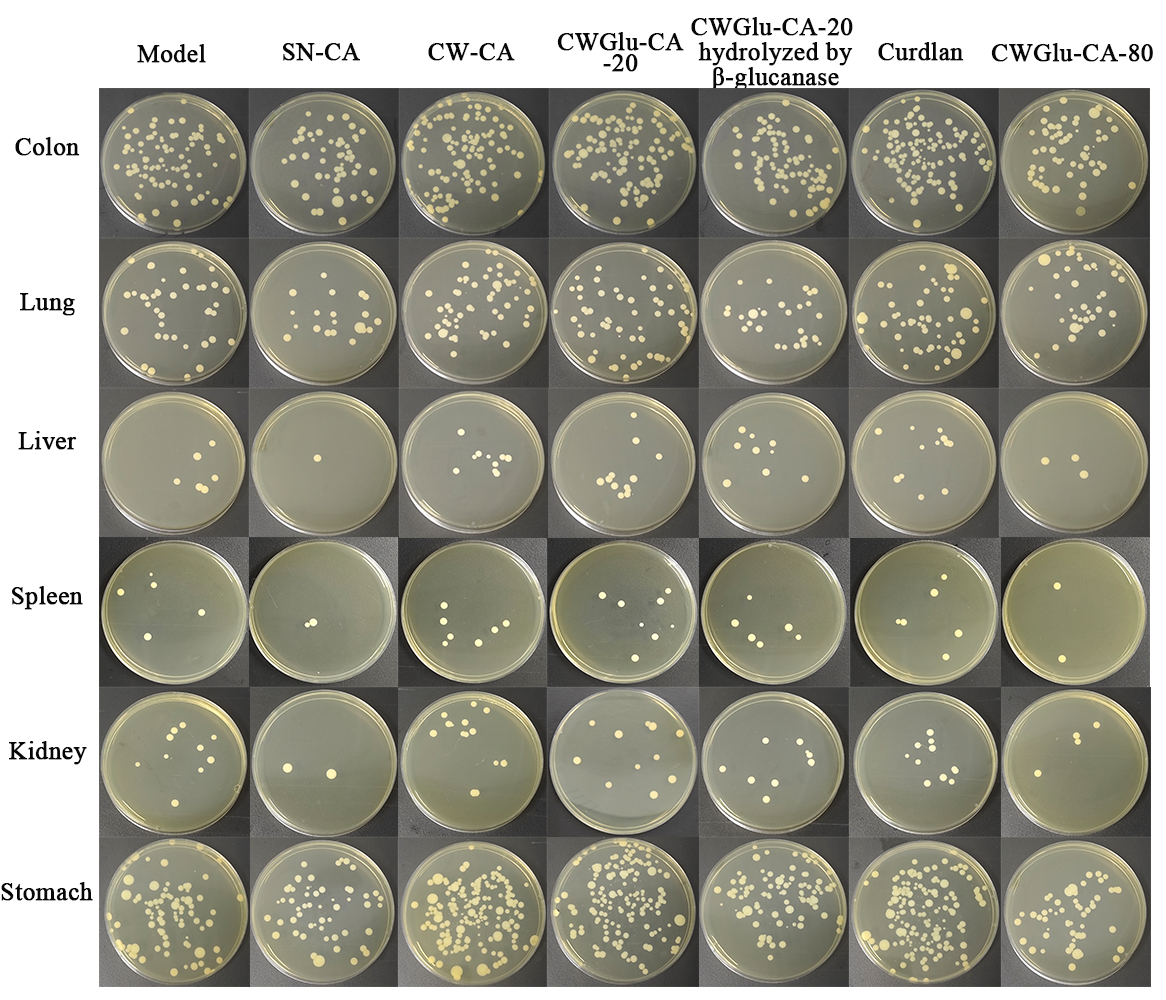

Supplement: Figure S4.tif [file KVIR_A_2514789_SM6653.tif]

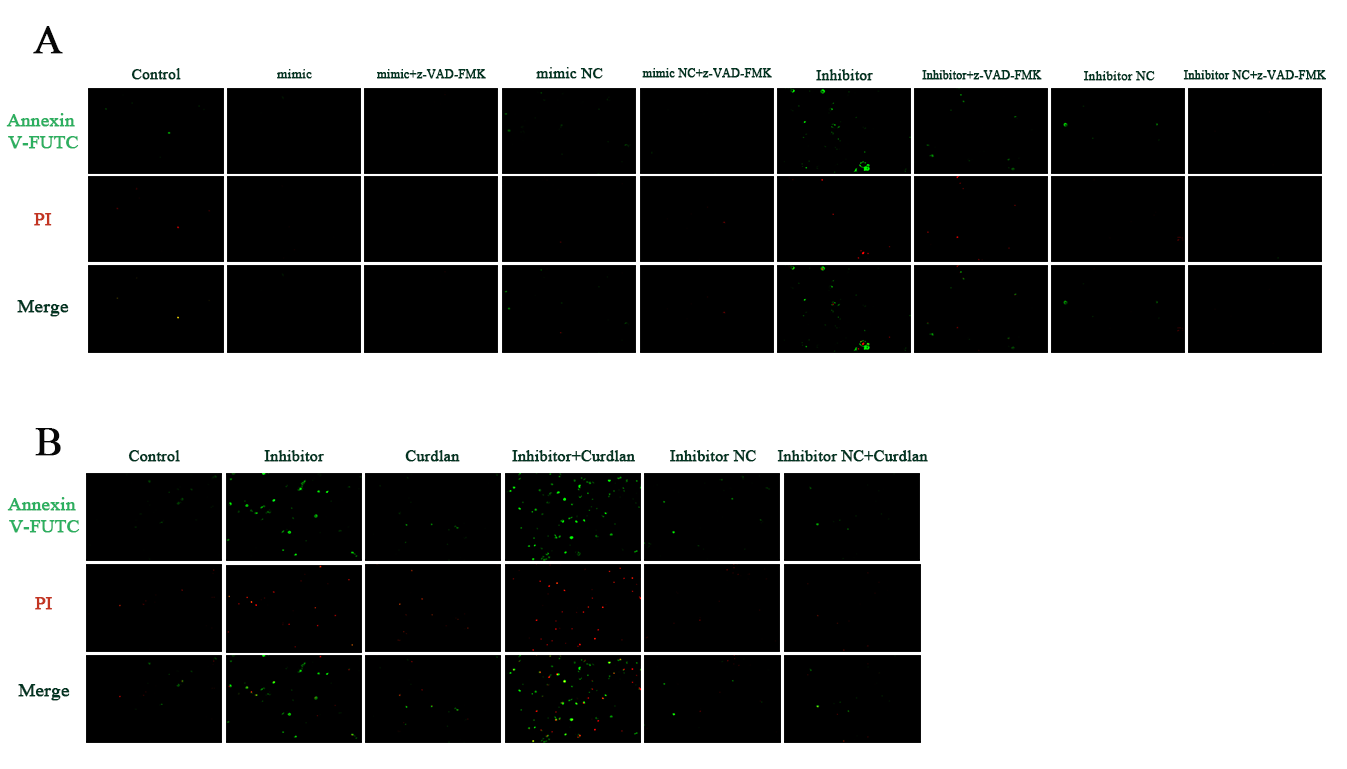

Supplement: Figure S7.tif [file KVIR_A_2514789_SM6652.tif]

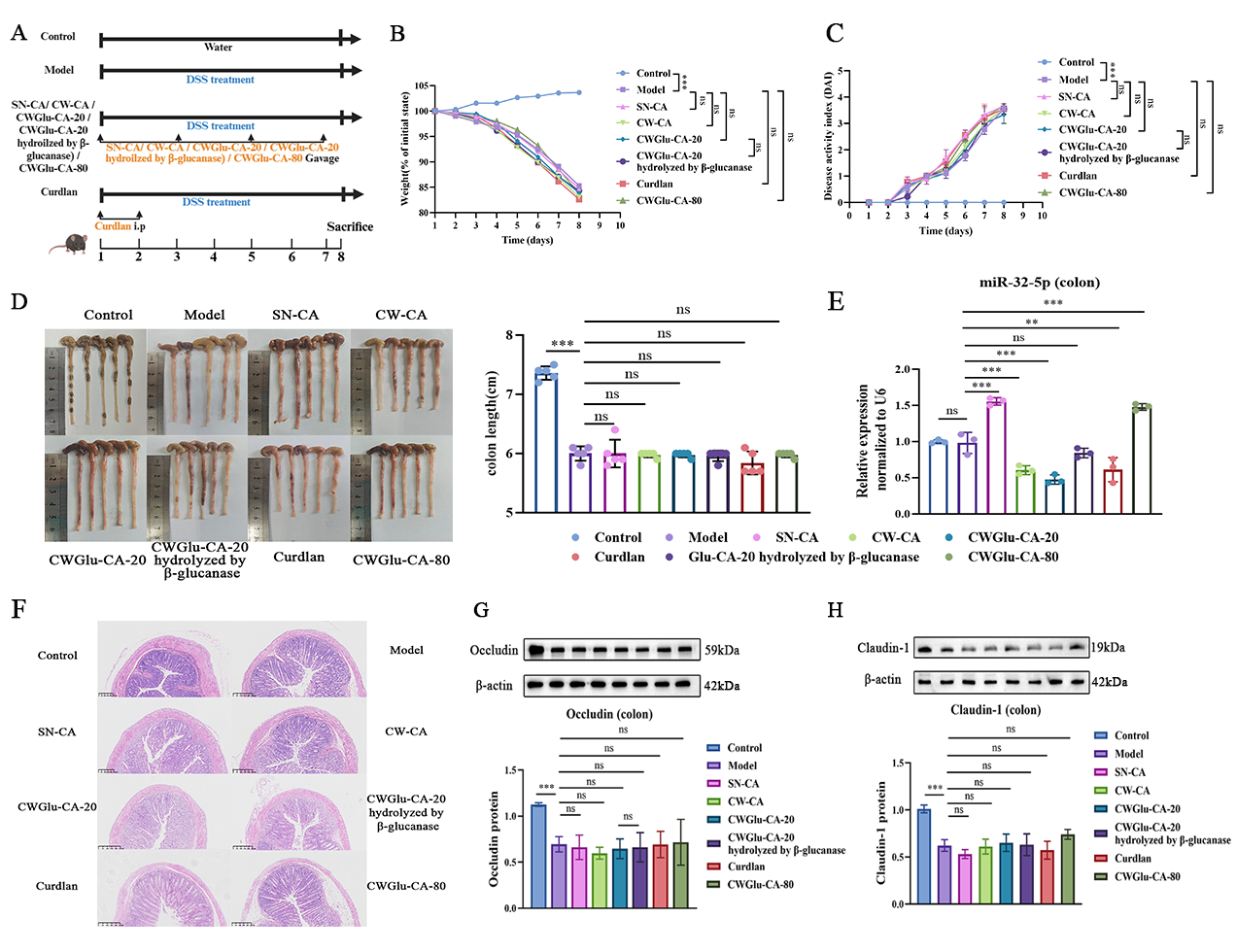

Supplement: Figure S5.tif [file KVIR_A_2514789_SM6651.tif]

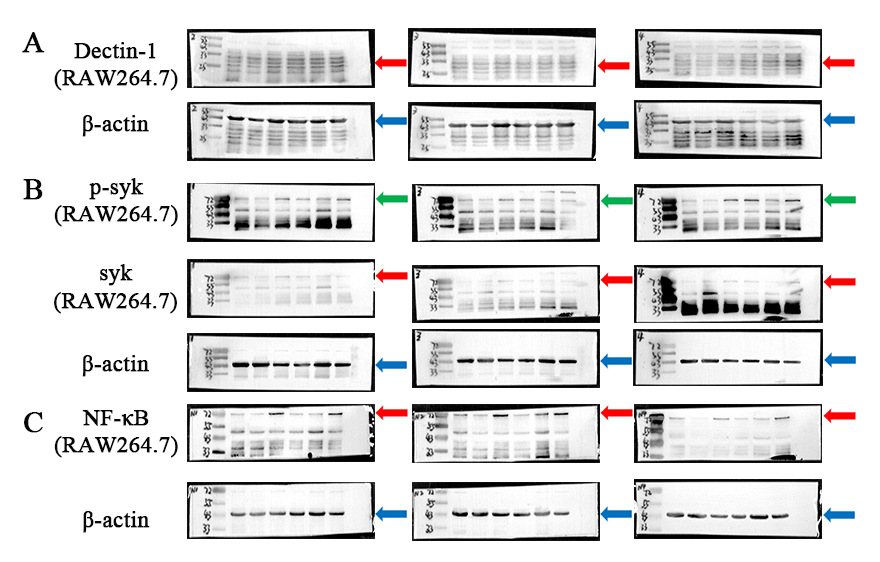

Supplement: Figure S8 4.tif [file KVIR_A_2514789_SM6650.tif]

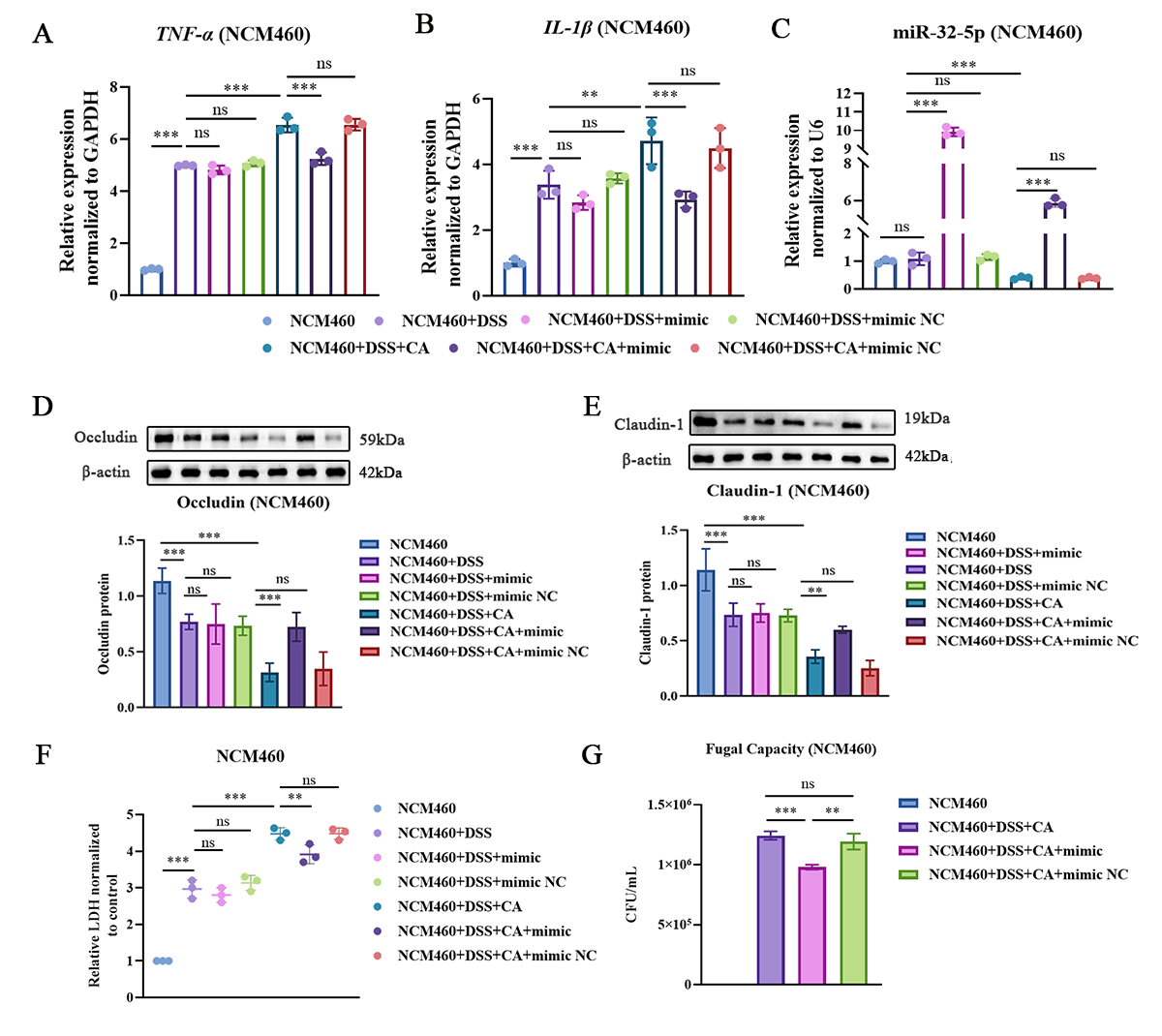

Supplement: Figure S1.tif [file KVIR_A_2514789_SM6649.tif]

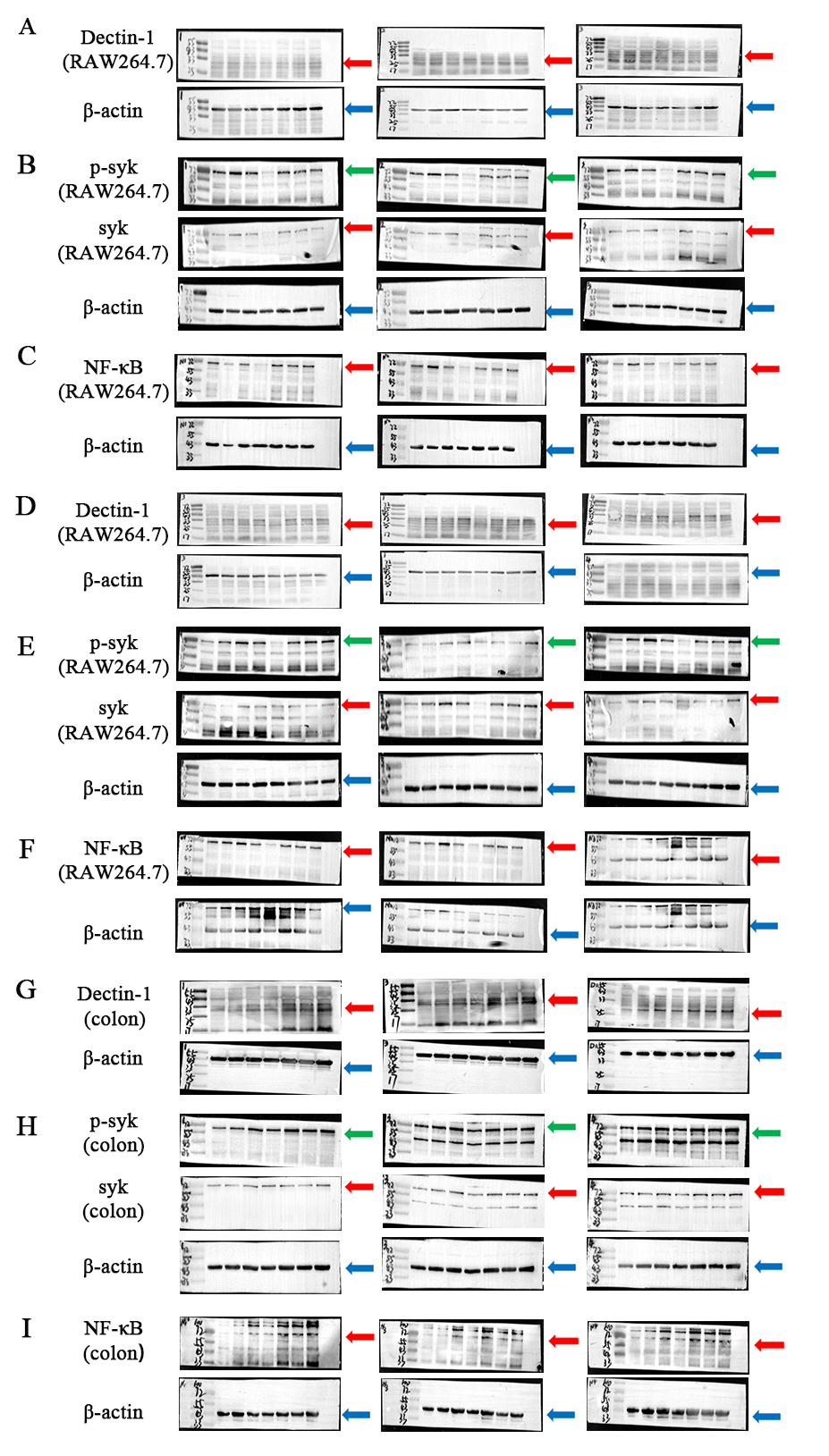

Supplement: Figure S8 3.tif [file KVIR_A_2514789_SM6648.tif]

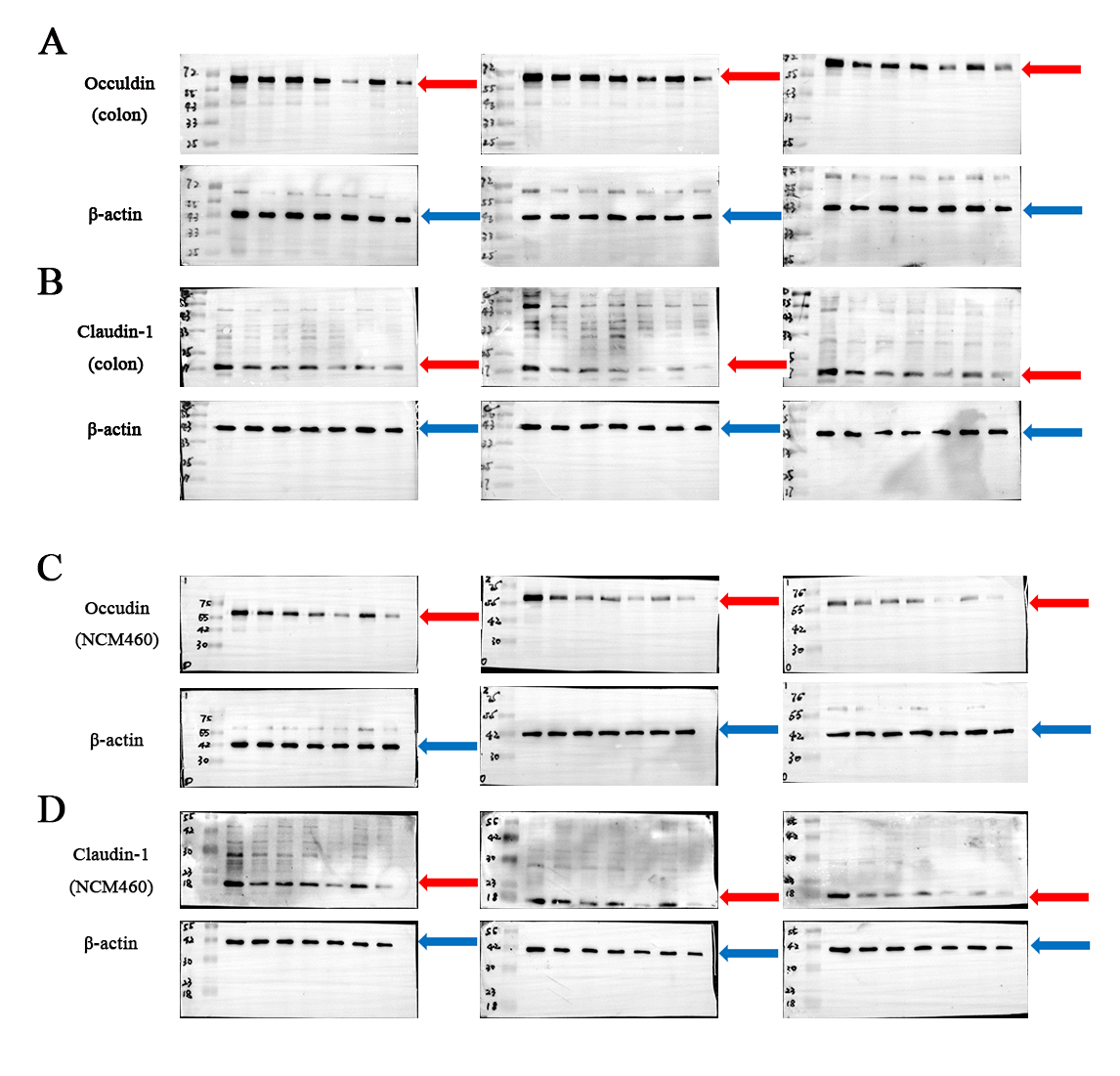

Supplement: Figure S8 1.tif [file KVIR_A_2514789_SM6647.tif]

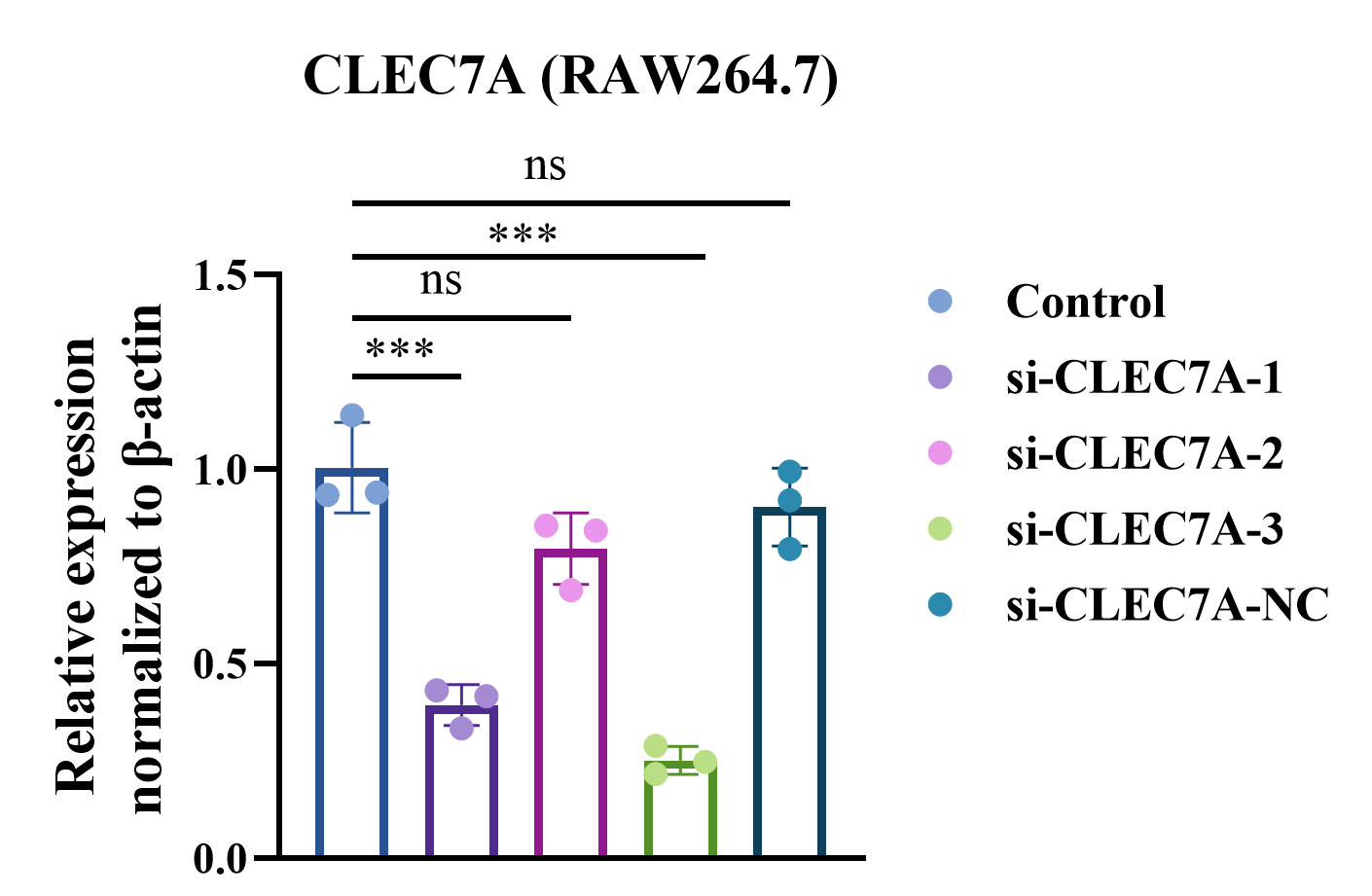

Supplement: Figure S6.tif [file KVIR_A_2514789_SM6646.tif]

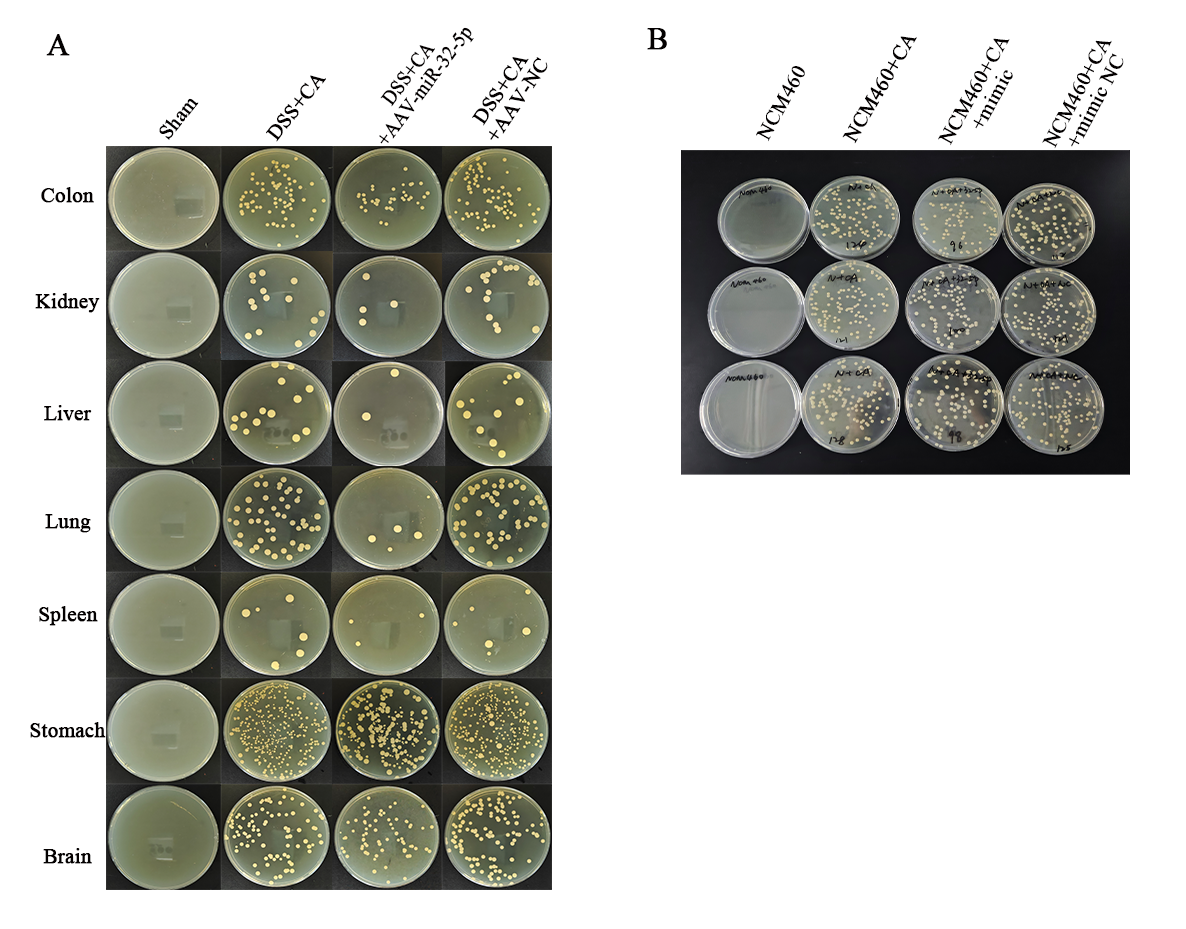

Supplement: Figure S2 - QVIR-2024-0854.R1.tif [file KVIR_A_2514789_SM6644.tif]

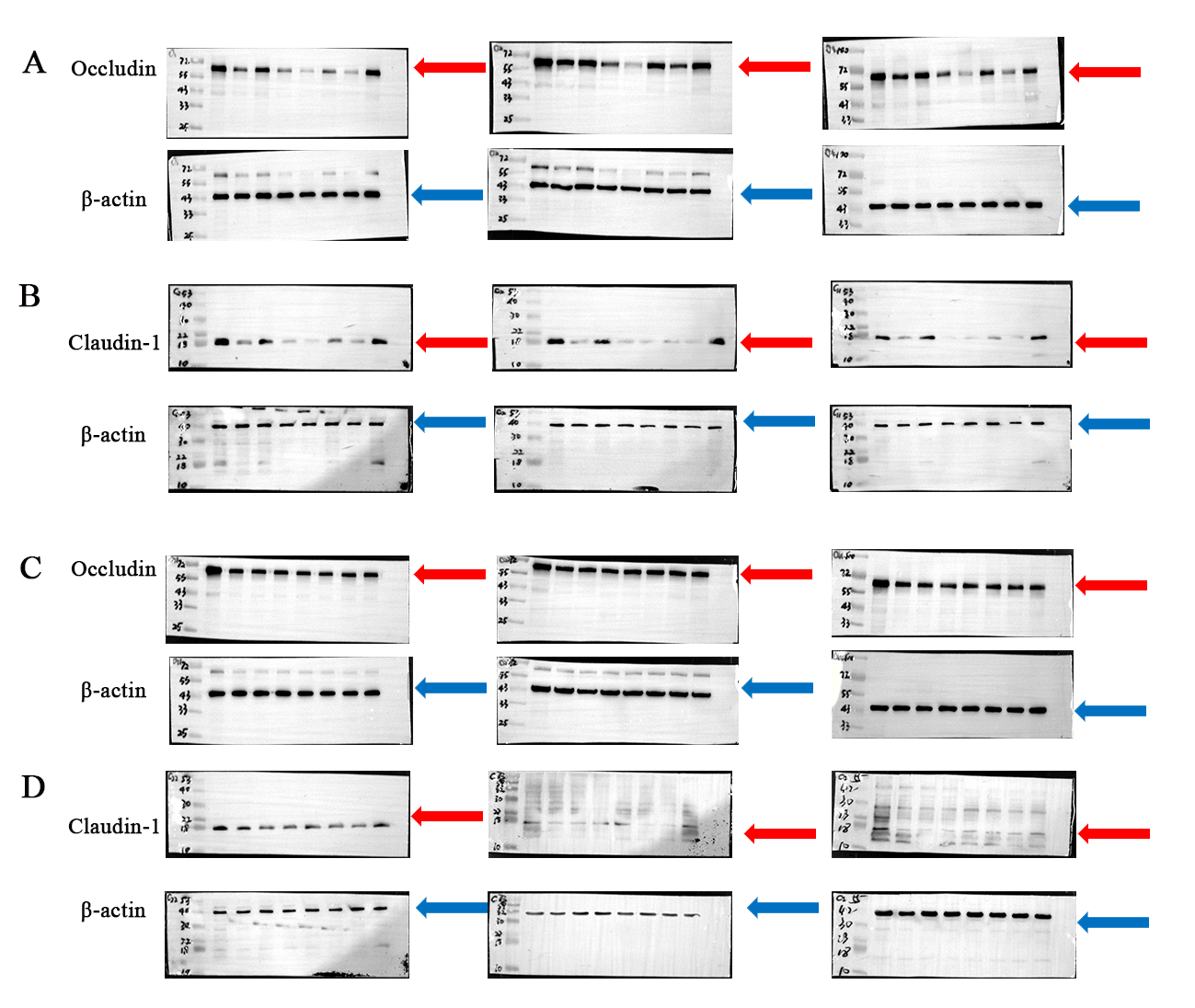

Supplement: Figure S8 2.tif [file KVIR_A_2514789_SM6643.tif]
